# Evolution of Hepatic Glucose Metabolism: Liver-Specific Glucokinase Deficiency Explained by Parallel Loss of the Gene for Glucokinase Regulatory Protein (GCKR)

**DOI:** 10.1371/journal.pone.0060896

**Published:** 2013-04-01

**Authors:** Zhao Yang Wang, Ling Jin, Huanran Tan, David M. Irwin

**Affiliations:** 1 Department of Laboratory Medicine and Pathobiology, University of Toronto, Toronto, Ontario, Canada; 2 Department of Pharmacology, Peking University, Health Science Center, Beijing, China; 3 Banting and Best Diabetes Centre, University of Toronto, Toronto, Ontario, Canada; Odense University Hospital, Denmark

## Abstract

**Background:**

Glucokinase (GCK) plays an important role in the regulation of carbohydrate metabolism. In the liver, phosphorylation of glucose to glucose-6-phosphate by GCK is the first step for both glycolysis and glycogen synthesis. However, some vertebrate species are deficient in GCK activity in the liver, despite containing *GCK* genes that appear to be compatible with function in their genomes. Glucokinase regulatory protein (GCKR) is the most important post-transcriptional regulator of GCK in the liver; it participates in the modulation of GCK activity and location depending upon changes in glucose levels. In experimental models, loss of GCKR has been shown to associate with reduced hepatic GCK protein levels and activity.

**Methodology/Principal Findings:**

*GCKR* genes and *GCKR*-like sequences were identified in the genomes of all vertebrate species with available genome sequences. The coding sequences of *GCKR* and *GCKR*-like genes were identified and aligned; base changes likely to disrupt coding potential or splicing were also identified.

**Conclusions/Significance:**

*GCKR* genes could not be found in the genomes of 9 vertebrate species, including all birds. In addition, in multiple mammalian genomes, whereas *GCKR*-like gene sequences could be identified, these genes could not predict a functional protein. Vertebrate species that were previously reported to be deficient in hepatic GCK activity were found to have deleted (birds and lizard) or mutated (mammals) *GCKR* genes. Our results suggest that mutation of the *GCKR* gene leads to hepatic GCK deficiency due to the loss of the stabilizing effect of GCKR.

## Introduction

Glucose, a major source of energy for all tissues, is obtained from the diet and stored as glycogen in the liver and muscle when in excess. Storage and release of glucose is a tightly regulated process involving numerous enzymes and regulatory proteins, with glucokinase (GCK) being a key regulatory enzyme [1]. GCK is an isozyme of the hexokinases, which catalyzes the phosphorylation of six-carbon sugars. GCK differs from the other hexokinases in its affinity for glucose and end-product inhibition [2]. GCK is the major glucose-phosphorylating enzyme in the liver, pancreatic islet beta-cells, and a few other glucose-sensing cells of the gut and brain [1]–[3]. In the liver, GCK is the first, and the rate-limiting, step in glucose utilization leading to glucose storage as glycogen, while in pancreatic beta-cells GCK acts as a glucose sensor and controls the secretion of insulin [2], [3]. GCK also appears to have similar glucose sensing functions in some cells of the gut and the brain [1], [3]. Mutations that prevent *GCK* expression or function in liver and pancreatic beta-cells are known to result in the maturity onset diabetes of the young 2 (MODY2) form of diabetes [4]. Similarly, activating mutations of *GCK* causing elevated GCK activity in liver and pancreatic beta-cells are also known to result in persistent hyperinsulinemic hypoglycemia of infancy (HPPI), in which high insulin levels induce low blood glucose levels [5].

A pair of tissue-specific promoters drives the expression of the *GCK* gene: an upstream beta-cell specific promoter that is also used in gut and neuronal cells; and a downstream liver-specific promoter [3], [6]. Liver-specific *GCK* expression is absolutely dependent upon the presence of insulin and is repressed by glucagon [3], [6]–[8]. Consequently, *GCK* expression is observed to increase after feeding or with insulin treatment, and expression is repressed when starved or with insulin deficiency [7], [8]. Hormonal regulation allows GCK to be active only when there is excess blood glucose, but the insulin effect on GCK is not potentiated by high glucose concentration [3], [6]–[8], in fact, glucose represses *GCK* expression, potentially to protect phosphate homeostasis [9]. In contrast to the nutritional regulation of *GCK* gene expression in the liver, little change in *GCK* mRNA levels is seen with changes in blood glucose levels or insulin in pancreatic beta-cells, although some change in mRNA stability might occur [3], [10]. The differences in the regulation of *GCK* expression in these two major sites of expression are likely related to the tissue-specific functions: whereas the liver only needs *GCK* expression when there is excess blood glucose, pancreatic islet beta-cells requires constant expression of *GCK* as a sensor for measuring blood glucose levels [2], [3].

In addition to regulation at the transcriptional level, GCK is also regulated in the liver at the post-transcriptional level by glucokinase regulatory protein (GCKR) [11], [12]. GCKR is primarily expressed in the liver [13], [14], and possibly the brain [15], with little if any expression in pancreatic beta-cells [16], [17]. In the liver GCKR functions as both an inhibitor and a nuclear binding protein for GCK [11], [18], [19]. Under low glucose conditions, GCKR binds, inactivates and sequesters cytosolic GCK into the nucleus. With an influx of glucose, GCK within the nucleus is re-activated and returns to the cytoplasm [20]–[22]. Phosphorylated forms of fructose, whose levels reflect glucose metabolism, modulate the binding of GCK with GCKR – fructose-1-phosphate weakens GCK and GCKR interaction, while fructose-6-phosphate promotes GCKR-GCK binding and subsequent GCK inactivation [3], [12], [23]. GCKR also has a role in stabilizing GCK and preventing its degradation [20]–[22]. Mice deficient in GCKR have normal or raised *GCK* mRNA levels but decreased liver GCK protein concentration and activity [24], [25]. Furthermore, overexpression of *GCKR* in hepatocytes by adenoviral vectors increases both GCK protein and enzymatic activity [26]. These observations suggest that the abundance of GCKR may be an important regulator of GCK protein levels specifically in the liver.

The livers of some mammalian (e.g., cow and cat) and non-mammalian (e.g., birds and lizards) vertebrate species have been reported to be deficient in GCK activity [27]. Hexokinases have been examined from the livers of numerous vertebrate species and their chromatographic profiles suggest that the livers of some mammals, including ruminants (e.g., cow and sheep), bats, and cats, as well as other vertebrate species such as birds and some reptiles, have little if any GCK activity [13], [27]–[31]. In these surveys, species deficient in GCK were found to possess activities of the three other hexokinases (i.e., hexokinases I, II, or III) at levels similar to those of species that have GCK [27]–[31] suggesting that the specific depletion of GCK in these species was not compensated by an increase in the activity of another hexokinase. As these species do not demonstrate symptoms of diabetes or poor glucose metabolism, they most likely possess an intact glucose sensing mechanisms; thus, mutations that inactivate GCK function seem unlikely. This conclusion is supported by our recent characterization of *GCK* genes from diverse species, where intact *GCK* genes were found in the genomes of most vertebrates examined [32]. An exception was the genome sequences of two bat species (flying fox and little brown bat), where the *GCK* genes were found to be missing their liver-specific first exon, which might prevent expression of *GCK* in the liver but not other sites [32].

As indicated above, mice with a targeted disruption of the *GCKR* gene show a specific deficiency of GCK activity in their liver [24], [25]. Low GCK activity is accompanied by an absence of GCKR in the liver of cats [33]. Since *GCKR* is the most important post-transcriptional regulator of GCK in most species and expressed almost exclusively in the liver of vertebrates that express GCK, including amphibians and reptiles [13], and not in the pancreatic beta-cells [16], [17], the other major site of GCK function, these observations suggest that the loss of GCKR may explain a liver-specific GCK deficiency. *GCKR* genes, though, have only been identified or characterized in a few species [34]–[36]. A previous evolutionary analysis of *GCKR* sequences identified *GCKR*-like genes in mammals, amphibians, and fish, as well as in non-vertebrate species, and concluded that *GCKR* evolved from an N-acetylmuramate 6-phospate esterase by changing its binding specificity and losing its esterase activity [36]. To address the hypothesis that liver-specific GCK deficiency is caused by the loss of GCKR, we identified and characterized *GCKR* genes from the genomes of diverse vertebrate species. Our results show that species deficient in liver GCK activity have mutated or deleted *GCKR* genes, supporting the hypothesis that the loss of GCKR, and its ability to stabilize GCK, explains the loss of liver-specific GCK activity.

## Materials and Methods

### Genome sequence data

Genomic sequences encoding GCKR-like protein sequences were downloaded from release 69 of the Ensembl and PreEnsembl databases (www.ensembl.org and pre.ensembl.org) in August 2012. Genomes from all available vertebrate species maintained in these databases were searched by gene name, gene symbol (*GCKR*), or by similarity searches with the tblastn algorithm [37] using the human GCKR protein sequence [35]. Searches of the non-redundant and genome databases maintained at the National Center for Biotechnology Information (NCBI, www.ncbi.nlm.nih.gov) were used to complement the genomes gathered from the Ensembl database and aided in the identification of incomplete genes or coding region sequences. Sequences similar to *GCKR* were not found in several species. For these species, genomic sequences for genes in the predicted conserved genomic neighborhood were included in this analysis. Genomes were searched for orthologs of the genes that flank the *GCKR* genes in diverse species (see results for details) and the genomic sequences adjacent to these genes were searched for similarity to *GCKR* genes.

### Alignment of sequences

Long genomic DNA sequences that included the *GCKR* gene were aligned with MultiPipMaker (pipmaker.bx.psu.edu/pipmaker/) [38], [39]. Human and mouse *GCKR* genes were used as masters for these alignments, and the locations of exons and coding regions for these genes was obtained from the Ensembl or NCBI databases. The identity and locations of repetitive elements in the human and mouse genomic sequences were identified using RepeatMasker (www.repeatmasker.org). Genomic alignments were used to refine the predicted potential coding regions of the genes. Potential pseudogenes were identified as sequences that failed to predict open reading frames due to the presence of base changes that either introduced stop codons, created frame shifts that disrupted the coding sequence, or disrupted splicing consensus sequences. Predicted open reading frames and protein sequences were aligned with the ClustalW algorithm [40] as implemented in MEGA5 [41], with the protein sequences used as guides for the nucleotide sequence alignments.

### Phylogenetic analysis

The origin of mutations that yielded the deletion of *GCKR* genes, or inactivation due to frame shift and/or splice site mutations were inferred by parsimony on the consensus vertebrate phylogenetic tree from Ensembl (www.ensembl.org). Phylogenetic trees were generated from the aligned GCKR protein sequences (see Figure S1 for alignment) using the neighbor joining method with Jones-Thornton-Thompson (JTT) or Dayhoff distances estimated using MEGA5 [41]. Phylogenetic trees of mammalian *GCKR* coding sequences were also generated from the DNA sequence data using either maximum likelihood composite distances or divergence at synonymous and nonsynonymous sites corrected by the Kimura 2-parameter method using MEGA5 [41]. The reliability of the trees was assessed by the bootstrap method, with the tree of vertebrate sequences rooted with the lamprey sequence and those of placental mammals with the sequence from a marsupial, the Tasmanian devil. Comparisons of the rates of sequence evolution on different species lineages were conducted using relative rate tests [42] as implemented in MEGA5 [41]. For relative rate tests, the sequence from the Tasmanian devil was used as the outgroup, and the numbers of unique amino acid substitutions was counted for each lineage. The probability that numbers of unique amino acid substitutions were equal was tested by a chi square test [42].

## Results

### Identification of *GCKR*-like genomic sequences

The human *GCKR* gene is composed of 19 exons distributed over roughly 27 kb of genomic DNA [43]. Previously, cDNAs and predicted genes for *GCKR* have been identified or characterized in a few other species, such as the rat, dog, zebrafish, tetraodon, and *Xenopus laevis* [14], [34], [36], and evidence for GCKR protein activity was identified in a few additional species, such as chicken, trout, carp, and goldfish [44]. To better characterize *GCKR* genes in vertebrates we searched the available genomes in the Ensembl (or preEnsembl) and NCBI databases for sequences that potentially encode GCKR. Our searches resulted in the identification of *GCKR*-like sequences in most species (Table 1) including all species for which a *GCKR* cDNA/gene had previously been identified. In the *Xenopus tropicalis* genome, a genome that is still not completely assembled, we identified four short genomic scaffolds containing sequences highly similar to the previously characterized *Xenopus laevis GCKR* cDNA and could be combined to predict a near complete *GCKR* gene sequence with only a single exon (exon 15) missing (Table 1). In a few other species (i.e., mouse lemur, sloth, and Chinese softshell turtle, Table 1) multiple genomic sequences were also identified that were consistent with a single gene because no part of the *GCKR* gene was found twice. An exception was the marmoset, where as many as four copies of the *GCKR* gene may exist (Table 1).

**Table 1:** Genomic location of vertebrate *GCKR* genes.

Species	Common name	Chromosome/Contig	Coding region	Ensembl Protein ID
*Homo sapiens*	Human	Chr 2	27,719,709-27,746,554	ENSP00000264717
*Pan troglodytes*	Chimpanzee	Chr 2A	27,882,887-27,909,760	ENSPTRP00000020223
*Gorilla gorilla*	Gorilla	Chr 2a	27,973,322-28,000,831	ENSGGOP00000012482
*Pongo abelii*	Orangutan	Chr 2a	84,605,694-84,632,388	ENSPPYP00000014009
*Nomascus leucogenys*	Gibbon	GL397331.1	6,798,770-6,825,699	ENSNLEP00000004669
*Papio hamadryas*	Baboon	Contig312330_Contig468503^1^	252,057-274,688	ENSP00000264717_1
*Macaca mulatta*	Macaque	Chr 13	27,491,384-27,514,309	ENSETEP00000001256
*Saimiri boliviensis boliviensis*	Bolivian squirrel monkey	Scaffold JH378252.1^1^	3,026,699-3,052,421	ENSP00000264717_1
*Callithrix jacchus*	Marmoset	Chr 14	80,920,115-80,945,184	ENSCJAP00000001541
		Ch 14	8,215,977-82,166,805	ENSCJAP00000051637
		GL285667.1	14,860-21,407	ENSCJAP00000046477
		GL289329.1	2,701-8,499	none
*Tarsius syrichta*	Tarsier	scaffold_142375	1-6,848	none
*Otolemur garnettii*	Bushbaby	GL873541.1	18,431,947-18,458,466	ENSOGAP00000008281
*Microcebus murinus*	Mouse Lemur	GeneScaffold_1280	170,174-180,857	ENSMICP00000014537
		scaffold_9408	44,808-49,358	
*Mus musculus*	Mouse	Chr 5	31,599,954-31,629,673	ENSMUSP00000072084
*Rattus norvegicus*	Rat	Chr 6^1^	36,176,192-36,204,485	NCBI: NP_037252.1^2^
*Cricetulus griseus*	Chinese hamster	Scaffold JH000049^1^	3,040,662-3,062,034	ENSMUST00000072228_1
*Dipodomys ordii*	Kangaroo rat	GeneScaffold_1944	64,619-93,530	ENSDORP00000004770
*Cavia porcellus*	Guinea pig	scaffold_18	9,315,790-9,341,258	ENSCPOP00000007381
*Ictidomys tridecemlineatus*	Squirrel	Scaffold JH393303	5,080,559-5,101,892	ENSSTOP00000004530
*Ochotona princeps*	Pika	GeneScaffold_1353	89,382-113,541	ENSOPRP00000011814
*Oryctolagus cuniculus*	Rabbit	Chr 2	158,578,950-158,598,892	ENSOCUP00000011833
*Bos taurus*	Cow	Chr 11	72,154,947-72,176,344	ENSBTAP00000041859
*Ovis aries*	Sheep	Chr 3^1^	36,829,349-36,855,060	none
*Tursiops truncatus*	Dolphin	GeneScaffold_1519	243,720-266,976	ENSTTRP00000011887
*Sus scrofa*	Pig	Chr 3	118,796,212-118,823,097	ENSSSCP00000020241
*Vicugna pacos*	Alpaca	GeneScaffold_2392	230,396-239,542	ENSVPAP00000001840
*Equus caballus*	Horse	Chr 15	68,799,209-68,819,071	ENSECAP00000018966
*Canis familiaris*	Dog	Chr 17	21,434,251-21,459,913	ENSCAFP00000007696
*Feli scatus*	Cat	Chr A3^1^	120,477,725-120,487,528	none
*Mustela putorius furo*	Ferret	Scaffold GL897010.1^1^	4,462,918-4,658,018	ENSCAFP00000009165
*Ailuropoda melanoleuca*	Panda	GL192369.1	463,160-487,028	ENSAMEP00000001336
*Myotis lucifugus*	Little brown bat	GL430052	860,474-888,947	none
*Pteropus vampyrus*	Flying fox bat	GeneScaffold_1035	519,562-527,386	ENSPVAP00000014342
*Tupaia belangeri*	Tree shrew	GeneScaffold_1539	218,507-244,816	ENSTBEP00000003453
*Erinaceus europaeus*	Hedgehog	GeneScaffold_2521	113,709-138,867	ENSEEUP00000006890
*Sorex araneus*	Shrew	Not found		none
*Loxodonta africana*	Elephant	scaffold_20	41,160,463-41,182,623	ENSLAFP00000005668
*Procavia capensis*	Hyrax	GeneScaffold_2165	16,762-42,556	ENSPCAP00000015899
*Echinops telfairi*	Lesse hedgehog tenrec	GeneScaffold_9143	353,017-423,957	ENSETEP00000001256
*Dasypu snovemcinctus*	Armadillo	GeneScaffold_3428	67,859-94,885	ENSDNOP00000014416
*Choloepus hoffmanni*	Sloth	GeneScaffold_3794	42,395-42,809	none
		scaffold_170360	376-835	
		scaffold_83977	28-3734	
*Monodelphi sdomestica*	Opossum	Chr 1	508,848,942-508,873,322	ENSMODP00000019482
*Macropus eugenii*	Wallaby	GeneScaffold_1196	275-7,845	ENSMEUP00000006501
*Sarcophilus harrisii*	Tasmanian devil	GL856719.1	1,420,689-1,458,490	ENSSHAP00000016711
*Ornithorhynchus anatinus*	Platypus	Not found		none
*Anolis carolinensis*	Anole Lizard	Not found		none
*Chrysemys pictabellii*	Painted turtle	JH585650.1^1^	8,656-43,647	none
*Pelodiscus sinensis*	Chinese softshell turtle	Scaffold JH210441.1	6,448-7,388	none
		Scaffold JH211948.1	132,429-114,901	
*Gallus gallus*	Chicken	Not found^1^		none
*Meleagris gallopavo*	Turkey	Not found		none
*Taeniopygia guttata*	Zebra finch	Not found		none
*Melopsittacus undulatus*	Budgerigar	Not found^1^		none
*Anas platyrhynchos*	Duck	Not found^1^		none
*Xenopus tropicalis*	Western clawed frog	GL176436.1	2,467-2,564	ENSXETP00000060990
		GL175638.1	1,885-7,075	
		GL181463.1	68-7,426	
		GL175969.1	6,442-11,214	
*Xenopus laevis*	African clawed frog			NCBI: NP_001083276.1^2^
*Latimeria chalumnae*	Coelacanth	Scaffold JH127635.1	202,083-223,756	ENSLACP00000006889
*Gadus morhua*	Cod	Not found		none
*Takifugu rubripes*	Takifugu	scaffold_64	713,339-722,055	ENSTRUP00000032438
*Oryzias latipes*	Medaka	Chr 2	24,524,153-24,535,721	ENSORLP00000005539
*Gasterosteus aculeatus*	Stickleback	groupI	23,443,090-23,447,491	ENSGACP00000019720
*Tetraodo nnigroviridis*	Tetraodon	Chr 3	4,336,678-4,340,938	ENSTNIP00000017284
*Danio rerio*	Zebrafish	Chr 17	5,624,859-5,639,741	ENSDARP00000124274
*Oreochromis niloticus*	Tilipia	Scaffold GL831144.1	2,399,129-2,414,317	ENSONIP00000015155
*Xiphophorus maculatus*	Platyfish	JH556962.1^1^	106,171-111,589	none
*Lepisosteus oculatus*	Spotted Gar	LG1^1^	45,522,798-45,550,720	none
*Petromyzon marinus*	Lamprey	GL476557	253,805-279,336	ENSPMAP00000010620

1 Sequences identified/searched for in the PreEnsembl database (some represent newer genome assemblies than those in Ensembl)

2 Protein ID from the NCBI database

### Missing *GCKR*-like genes

While genomic sequences similar to *GCKR* were found in most vertebrates, sequences similar to *GCKR* were not found in the genome sequences of two mammals (shrew and platypus), a reptile (anole lizard), all 5 available birds (chicken, turkey, duck, zebra finch, and budgerigar), and a fish (cod) (Table 1). Since genome assemblies for some species are still at a draft stage it might be expected that the failure to identify a *GCKR*-like sequence may simply be due to gaps in the available genomes. In contrast, our searches of these same genomes for *GCK* gene-like sequences resulted in the identification of complete or partial genes in all of these mammalian [32] and non-mammalian vertebrate species (Table S1), suggesting that the absence of sequences similar to *GCKR* in at least some (but possibly not all) of the genomes is due to the loss of this gene. Gene loss can be due to either deletion of a sequence from the genome, or failure to find using the blast similarity search algorithm as the sequence became non-functional and degenerated into a pseudogene.

**Figure 1:**
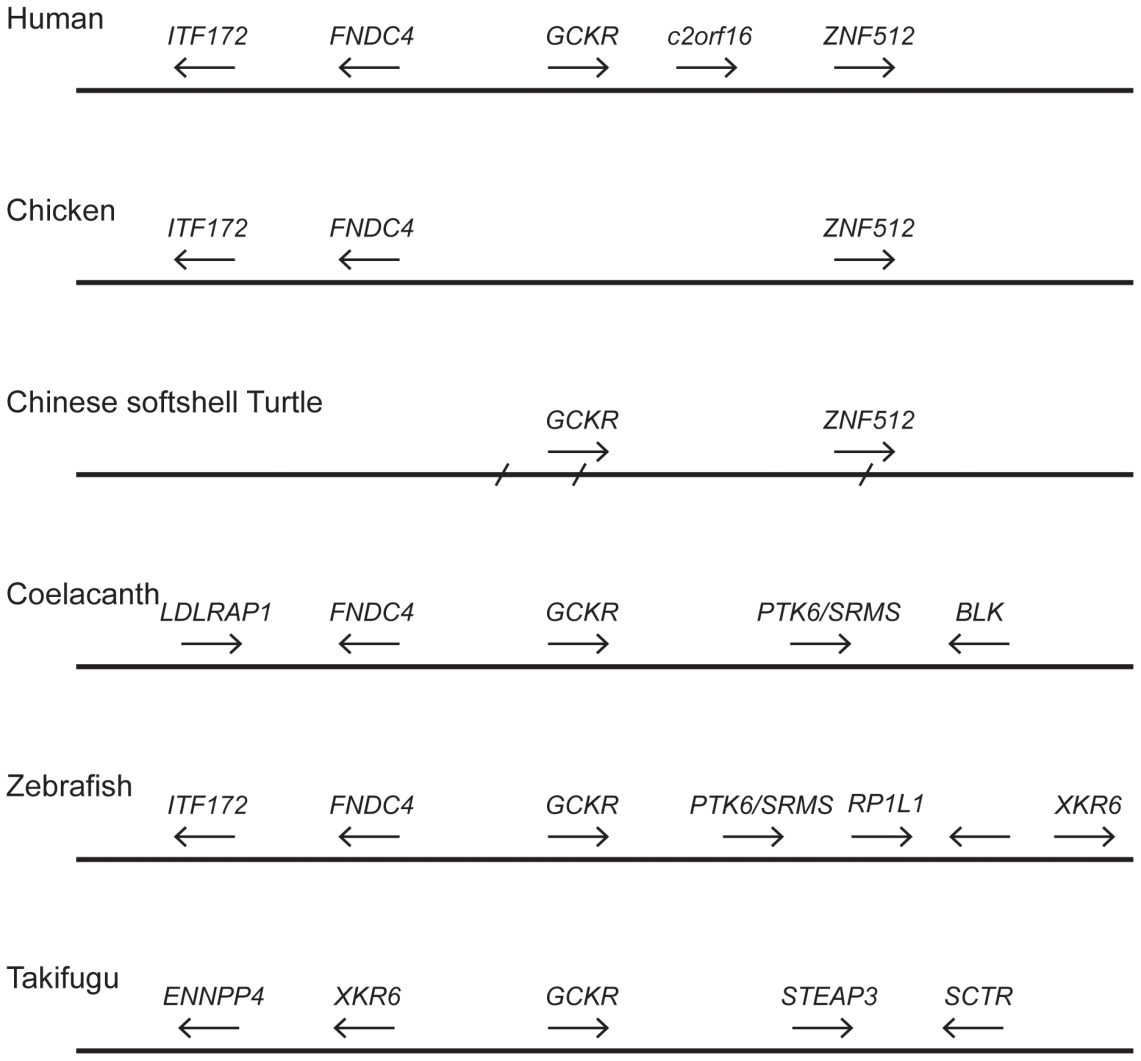
Genomic neighborhoods around vertebrate *GCKR* genes. Relative order and orientation of genes near the *GCKR* genes in human, chicken, Chinese softshell turtle, coelacanth, zebrafish and takifugu genomes. Gene names, as annotated in the human genome, are shown above the arrows, with the arrowhead indicating direction of transcription. Gene sizes and distance between genes are not to scale. Human is representative of the gene organization in mammals, while chicken is representative of birds. *GCKR* gene was not found in bird genomes. In fish, two distinct gene organizations were found – one found in zebrafish, and a second found in other fish genomes and represented by takifugu. The Chinese softshell turtle GCKR gene was distributed over two genomic contigs, with the slashes (/) indicating the ends of contigs.

Missing genes can sometimes be found if the gene order surrounding a gene is conserved, as the adjacent genes can then be used to search for orthologous genomic regions. This approach has successfully been used to identify genes from divergent genomes showing minimal or no similarity in blast searches, such as a homolog of the mammalian leptin gene in fish [45]. To determine whether this approach might be useful, we examined the gene neighborhoods flanking the *GCKR* genes to determine whether gene order is conserved in vertebrate genomes. The genes *FNDC4* (fibronectin type III domain containing 4) and *ITF172* (intraflagellar transport 172 homolog) are located 5′ to the human *GCKR* gene, while *ZNF512* (zinc finger gene 512) gene and the predicted gene *C2orf16* (chromosome 2 open reading frame 16) are located 3′ (Figure 1). Genomes of other mammals possessing the *GCKR* gene have the same gene order – for some species, the genomic fragment containing *GCKR* is shorter and do not predict all four genes. While a *GCKR* gene could not be found in any of the bird genomes, it was found in two of the three reptile genomes, albeit they were on short genomic fragments (Table 1). Searches using the Painted turtle genomic sequence failed to identify any additional gene; however, a sequence downstream of the 3′ end of the Chinese softshell turtle *GCKR* gene is similar to the *ZNF512* gene (Figure 1). The *ZNF512* gene is located 3′ and in the same transcriptional orientation as *GCKR* in both mammals and a turtle (Figure 1), suggesting that this order existed in their common ancestor.

The *Xenopus tropicalis GCKR* gene is distributed over four small genomic fragments (Table 1) that do not show similarity to any other gene. The coelacanth is a lobe-finned fish that is more closely related to tetrapods (mammals, birds, reptiles and amphibians) than to other fish [46]. Genes similar to *FNDC4* and *LDLRAP1* (low density lipoprotein receptor adaptor protein 1) are found 5′ to the coelacanth *GCKR* gene, while genes similar to *PTK6*/*SRMS* (protein tyrosine kinase 6/src-related kinase lacking C-terminal regulatory tyrosine and N-terminal myristylation sites) and *BLK* (B lymphoid tyrosine kinase) were found 3′ (Figure 1). The gene neighborhood near the coelacanth *GCKR* gene differs considerably from that of the human *GCKR* gene, this suggests that recombination has occurred; however, the *FNDC4* is located in a similar position with the same transcriptional orientation, in both species, suggesting that the genomic linkage of these two genes was present in their common ancestor.

The gene neighborhood of the zebrafish *GCKR* gene strengthens the conclusion of an ancestral *GCKR* – *FDNC4* genomic linkage, as the zebrafish also has an *FNDC4*-like gene 5′ to its *GCKR* gene (Figure 1). The zebrafish genome also predicts an *IFT172*-like gene in a position orthologous to the human *IFT172* gene, and a *PTK6*/*SRMS6*-like gene in a position orthologous to that found in the coelacanth genome (Figure 1). These observations suggest that the *FNDC4* and *IFT172* genes were 5′ to the *GCKR* gene (as seen in the human and zebrafish genomes) and the *PTK6*/*SRMS* gene was located 3′ to *GCKR* (as seen in the zebrafish and coelacanth) in the common ancestor of fish and tetrapods. The gene order near the *GCKR* gene in the other fish genomes examined differed from that of zebrafish, and they shared an order that was consistent with that shown for the takifugu (Figure 1). All of the fish genomes have an *XKR6* (XK, Kell blood group complex subunit-related family, member 6) gene near the *GCKR* gene, albeit in different relative positions and in a different transcriptional orientation, suggesting that this genomic region has been reorganized within fish.

Based on these gene maps (Figure 1), we used the conserved flanking genes to identify orthologous genomic regions in species where Blast failed to identify a *GCKR*-like sequence. Since the ancestral tetrapod is predicted to have an *FNDC4*-like and a *ZNF512*-like gene flanking the *GCKR* gene, we used these sequences to search the mammalian genomes (shrew and platypus), reptilian (anole lizard) and avian (chicken, turkey, duck, zebra finch and budgerigar) genomes where we failed to identify a *GCKR* gene with our similarity searches (see Table 1). Searches of the mammalian (shrew and platypus) and the reptilian (anole lizard) genomes resulted in identifying only short genomic fragments encoding sequences similar to *FNDC4* and *ZNF512*, with none of these genomic fragments encoding any additional genes (results not shown). This result is consistent with the low coverage and fragmented nature of these genome sequences, and thus we cannot determine whether the gene neighborhood is conserved, or if the *GCKR* gene was lost or is present in an unsequenced gap of these genome assemblies. In contrast, large genomic fragments were typically identified from the avian genomes, where the *FNDC4* and *ZNF512* genes were linked on the same genomic fragment, as shown for the chicken in Figure 1. Searches of the genomic sequences between the *FNDC4* and *ZNF512*-like genes from the bird genomes failed to identify any sequence with similarity to *GCKR*. These results strongly suggest that the *GCKR* gene was deleted from the avian genomes, and that this deletion event occurred before the divergence of the avian lineages represented by the genome sequences. The cod genome was also searched with genes that flank the *GCKR* gene in different fish genomes, and a genomic fragment with some similarity to that seen in coelacanth was found (containing *FNDC*, *LDLRAP1*, and *BLK*-like genes), but different from other ray-finned fish. This suggests that further reorganization has occurred (results not shown). Due to recombination, it is difficult to determine whether the cod *GCKR* gene was lost or is at a different location, such as an unsequenced gap, in this genome.

### *GCKR* pseudogenes

Surprisingly, *GCKR* genes from many species, especially mammals, are poorly annotated (i.e., truncated or missing exons) or not annotated at all in the Ensembl database. To determine why some *GCKR* genes are poorly annotated we used MultiPipMaker [38], [39] to align the genomic sequences of mammalian *GCKR* genes to the well-characterized human *GCKR* gene [43]. With the human exon sequences as guides, coding exons in the other mammalian *GCKR* genes could be identified. Similar results were obtained when the mouse *GCKR* gene was used as the master sequence for the MultiPipMaker analysis (results not shown). Alignments of the *GCKR* gene exons allowed us to identify changes that have occurred in the *GCKR* gene in each species. Many of the exons that had not been annotated as exons of the *GCKR* genes in species with poorly annotated *GCKR* genes in the Ensembl database were found to have mutations that either introduced frame shifts (due to insertion or deletion of sequences) into the coding sequence, or would be predicted to prevent mRNA splicing (Table 2). The effect of these mutations likely explains why gene prediction programs that annotated these genome assemblies failed to identify these sequences as coding exons (since splicing and coding potential are properties that should be retained by coding exons). The *GCKR* genes of many species were found to contain multiple disrupting mutations. In some cases, more than half of the 19 coding exons (e.g., cow with 11 exons) harbored mutations predicted to disrupt splicing or translation (Table 2). Only a few species (squirrel, tree shrew, and opossum) have a single disrupting mutation that potentially could be explained as a sequencing error or the sequence of a rare mutated allele present in the population of alleles for those species. A total of 14 mammalian species were identified to have *GCKR*-like genes in their genomes that likely do not encode a functional GCKR protein as they have two or more coding sequence disrupting mutations (Table 3).

**Table 2:** Exons bearing inactivating mutation in mammalian *GCKR* genes.

	Exons with mutations	
Species	Frame shift mutations	Splice junction mutations
Tarsier	10, 15, 16	16
Squirrel	8	–
Cow	2, 5, 7, 8, 15, 17, 18, 19	2, 9, 10, 12, 15, 18
Sheep	5, 6, 8, 12, 18	9, 10, 12
Dolphin	1, 3, 4, 6, 8, 9, 15, 19	1
Alpaca	5, 7	1
Cat	2, 4, 10	2
Ferret	–	9, 10, 12
Little brown bat	2	3
Flying fox bat	5, 6, 8, 10, 12	5, 8, 10, 11
Hedgehog	1, 3, 4, 17	2
Tree shrew	4	–
Hyrax	1, 3, 5, 10, 11, 17	1, 3, 4, 5, 7, 11, 19
Lesser hedgehog tenrec	5, 7, 8, 9, 13, 17, 18	5, 6, 9, 13, 14, 15
Armadillo	8, 13, 14, 15, 19	13, 15, 16, 18, 19
Sloth	7, 13, 19	2, 4, 5, 6, 7, 18
Opossum	12	–

**Table 3:** Vertebrate *GCKR* genes.

Intact coding regions					
Human		Chimpanzee	Gorilla	Orangutan	
Gibbon		Macaque	Baboon	Mouse lemur	
Mouse		Rat	Chinese hamster	Guinea pig	
Rabbit		Pig	Horse	Dog	
Panda		Elephant	Tasmanian devil	African clawed frog	
Spotted gar					
Potentially intact genes					
Squirrel monkey		Marmoset	Bushbaby	Kangaroo rat	
Squirrel*		Pika	Tree shrew*	Opossum*	
Wallaby		Painted turtle	Chinese softshell turtle	Western clawed frog	
Coelacanth		Takifugu	Medaka	Stickleback	
Tetraodon		Zebrafish	Tilapia	Platyfish	
Lamprey					
Genes with inactivating mutations					
Tarsier	Cow		Sheep		Dolphin
Alpaca	Cat		Ferret		Little brown bat
Flying fox bat	Hedgehog		Hyrax		Lesser hedgehog tenrec
Sloth	Armadillo				
Deleted genes					
Shrew	Platypus		Anole lizard		Chicken
Turkey	Zebra finch		Budgerigar		Duck
Cod					

* Predicted genes have a single frame shift mutation that may be a sequencing error.

The spotted gar was the only non-mammalian genome that contained a genomic sequence where all 19 *GCKR* coding exons could be identified and could be used to predict an intact GCKR protein (Table 1 and Figure S1). For all other non-mammalian species, the *GCKR*-like genomic sequence was missing at least one exon, most often exon 1. Unfortunately the missing exons cannot be identified because the non-mammalian (turtle, frog, and fish) genomic sequences do not align with the human, or any other mammalian, *GCKR* gene sequence used as a master sequence in MultiPipMaker (results not shown), due to the large amount of sequence divergence that has occurred since their common ancestor that existed at least 300 million years ago [47]. Similar results were obtained when the spotted gar sequence was used as master sequence, as it also is distantly related to all of the other species (including ray-finned fish, whom they diverged from more than 300 million years ago [48]). However, the coding exons that were identified for the non-mammalian *GCKR* genes (see Table 1) maintained open reading frames and possessed intact spice consensus sequences that should allow the generation of an intact GCKR proteins (see Figure S1). While it remains possible that inactivating mutations exist in some of the uncharacterized exons, our current genomic evidence suggests that functional GCKR proteins exit for all of the non-mammalian species where a *GCKR* gene was identified.

### Parallel inactivation of mammalian *GCKR* genes

**Figure 2:**
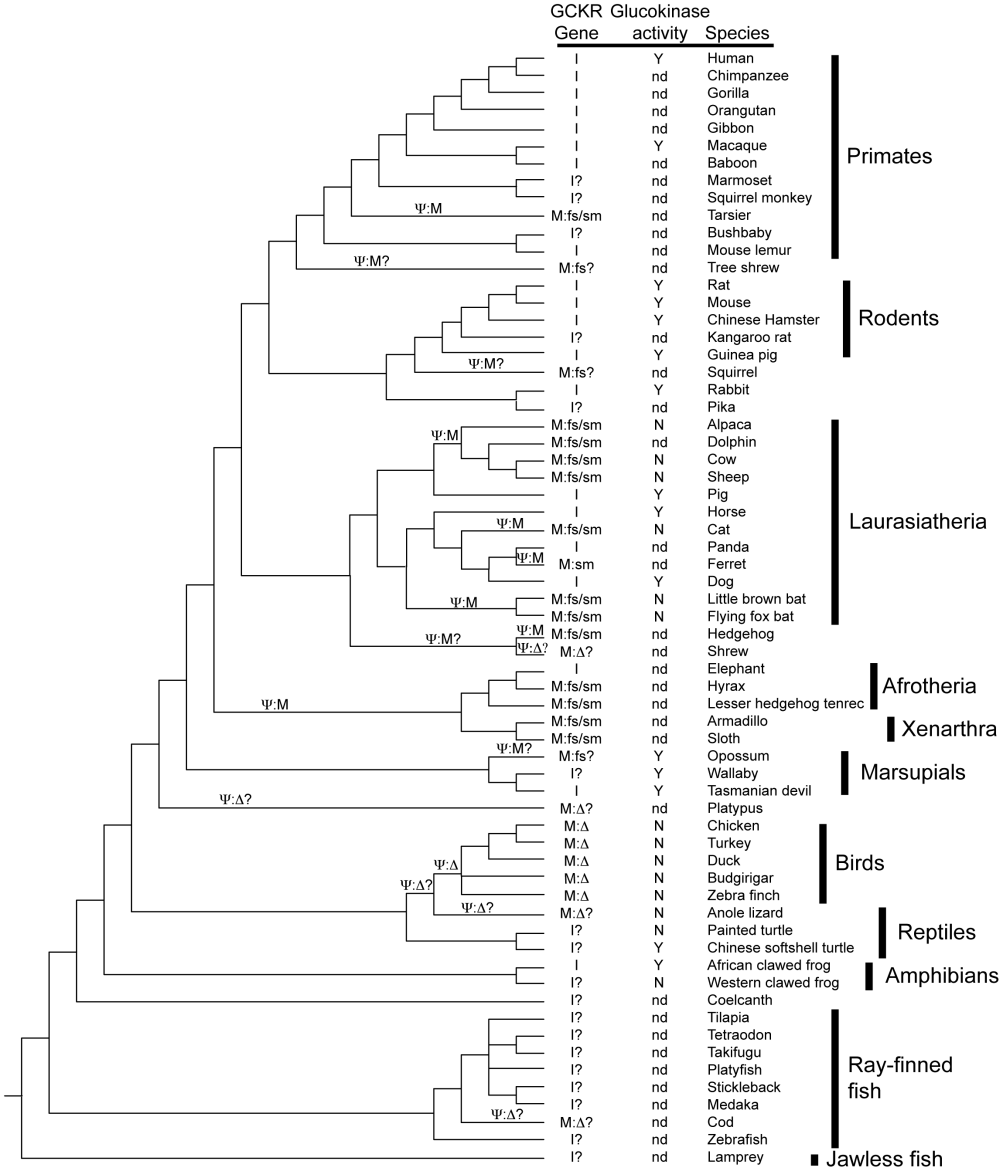
Evolution of *GCKR* genes and hepatic GCK activity in vertebrates. Summary of information on the structure of *GCKR* genes and hepatic GCK activity are placed on a phylogeny of vertebrates with available genome sequences (with the common names of the species shown on the right). Higher-level taxonomic groups of species are indicated to the right, with their composition indicated by the vertical bars. The phylogenetic relationship is from Ensembl (www.ensembl.org). *Xenopus laevis* is added to the tree as it has an intact *GCKR* cDNA. Genes are labeled as intact (I), likely intact (I?) or mutated (M), with the types of mutation indicated: fs = frame shift, sm = splice mutation, Δ = deletion, and Δ? = likely deletion (see Tables 2 and 3). The phylogenetic placement of gene inactivation events (Ψ, with type of inactivation indicated) was determined by parsimony. Possible inactivations, or events with unresolved locations (i.e., on the bird lineage), are indicated by the ? symbol. Hepatic GCK activity is from references 27-31, with Y = activity found, N = no or very low activity, and nd = not determined.

Our characterization of vertebrate *GCKR* genes indicates that functional versions of this gene has been lost by several mechanisms, including point mutations that disrupt splicing or cause translational frame shifts or deletion mutations which have removed the gene from the genome (Tables 2 and 3). Evidence for deletion of the gene is strongest in birds, while the failure to find a *GCKR* gene in some of the other species could be due to gaps in the current genome assemblies. All birds that have an assembled genome lack a *GCKR*-like gene sequence, suggesting that the gene was deleted before the common ancestor of birds (see Figure 2). The anole lizard is closely related to birds and lacks a *GCKR*-like gene (Table 1) suggesting that the deletion of the *GCKR* gene may have occurred in a reptilian ancestor of the anole lizard and birds (see Figure 2). The *GCKR* gene was not deleted in the common ancestor of all reptiles as the gene was found in turtle genomes (Table 1). Within mammals, inactivating mutations appears to be the dominant mechanism for the loss of *GCKR* gene function – 14 species showed inactivated genes due to mutations, while only 2 species may have deleted the gene (Table 3). A single mutational event cannot explain the origin of the inactivated mammalian *GCKR* genes. First, no single mutation is shared among the inactivated *GCKR* genes, even among the mutationally inactivated genes (see Table 2). Secondly, species with inactivated *GCKR* genes are not monophyletic (Figure 2). Parsimony was used to infer the minimum number of gene inactivation events on the phylogeny of vertebrates. At least six gene inactivation events are required to explain the diversity of species that have multiple mutations (i.e., at least one frame sift and at least one splice site mutation) (Figure 2). Inactivating mutations must have occurred in the *GCKR* gene on the lineages leading to: (1) tarsier, (2) ruminant artiodactyls, (3) cat, (4) bats, (5) hedgehog, and (6) common ancestor of Afrotheria and Xenarthra (see Figure 2).

### Are some intact *GCKR* genes pseudogenes?

**Figure 3:**
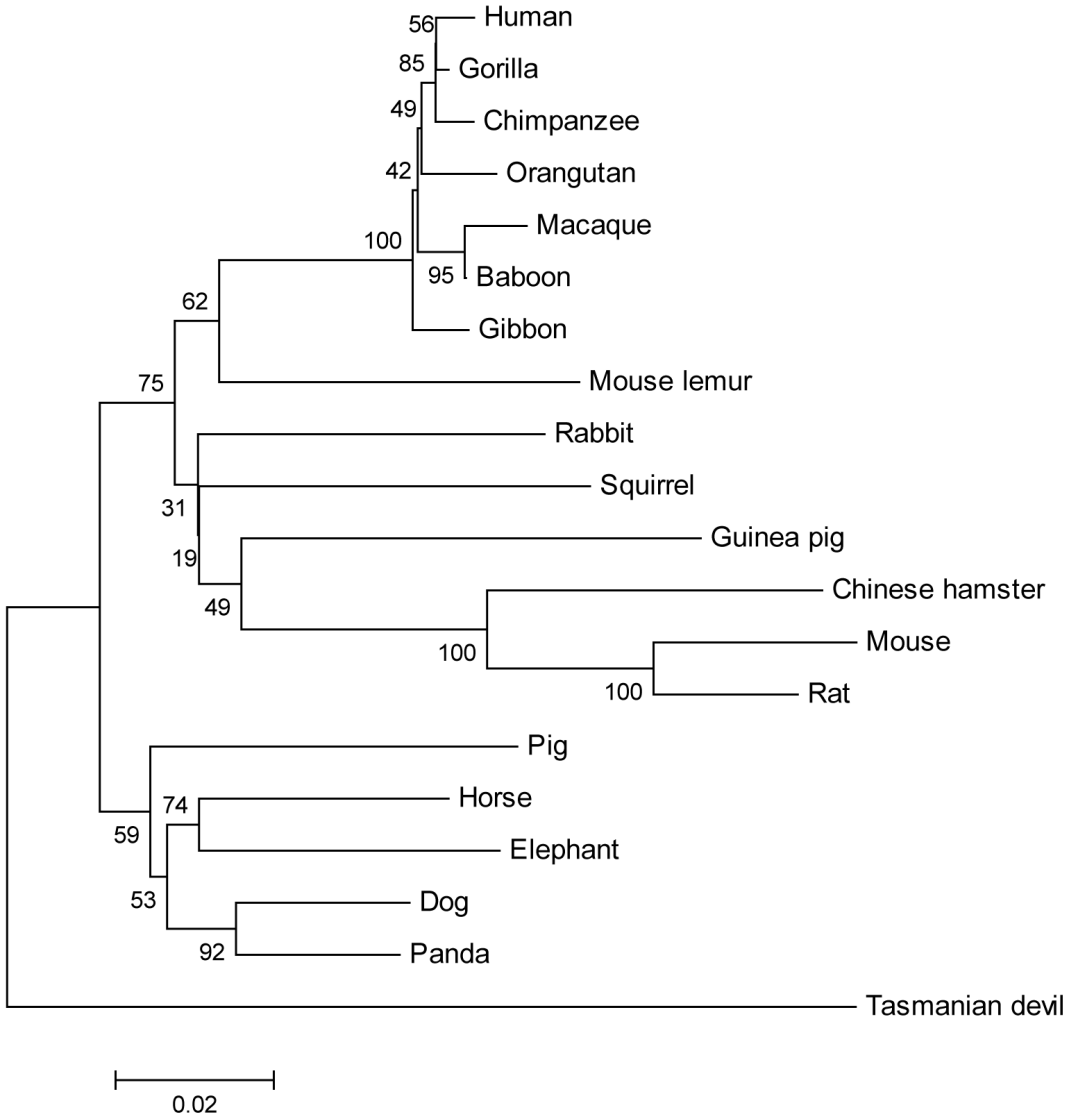
Phylogeny of mammalian GCKR protein sequences. Phylogeny of mammalian GCKR protein sequences generated from full length *GCKR* coding sequences (see Figure S1). The bootstrapped (1000 replications) neighbor-joining distance tree was generated using JTT protein distances. The sequence from the Tasmanian devil was used as the outgroup. Similar trees were generated when different protein distance measures, or distance measures based on nonsynonymous distances calculated from aligned DNA sequences, were used or if trees were built by other methods, such as parsimony or maximum likelihood.

**Figure 4:**
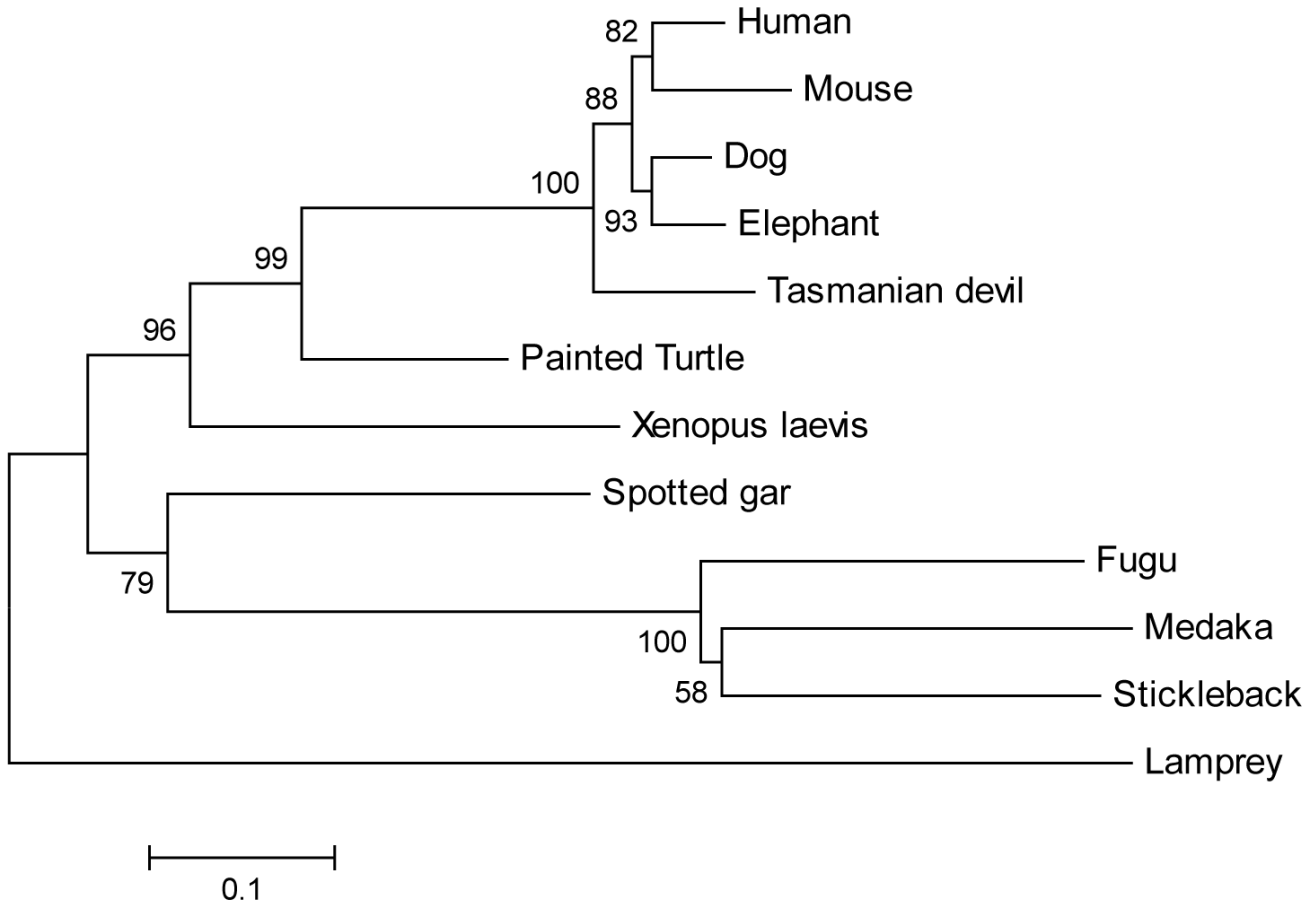
Phylogeny of vertebrate GCKR protein sequences. Phylogeny of vertebrate GCKR protein sequences generated from intact and near-full length *GCKR* coding sequences (see Figure S1). Only select mammals were included in the analysis. The bootstrapped (1000 replications) neighbor-joining distance tree was generated using JTT protein distances. The Lamprey (jawless fish) sequence was used as the outgroup. Similar trees were generated when different protein distance measures were used or if other tree building methods, such as parsimony or maximum likelihood, were used.

Many of the inactivated *GCKR* genes are found in Laurasiatheria (mammalian orders: Artiodactyls, Perisodactyls, Carnivores, Chiropteria, Eulipotyphla, and Pholidota) where 4 parallel gene loss events are inferred to have occurred (Figure 2). An alternative explanation for this distribution of inactivated genes is that all of the Laurasiatherian *GCKR* genes are pseudogenes, which were generated by a single mutational event that did not disrupt the open reading frame but did disrupt GCKR protein function. Subsequent to the ancestral inactivating mutational event, additional mutations introduced frame shifts and splice site mutation into the *GCKR* genes of most of the species representing Laurasiatheria (e.g., cow, cat, and bats). However, the gene sequences from dog, panda, horse, and pig did not acquire these types of mutations, and thus retained intact open reading frames. Pseudogenes should acquire greater numbers of mutations compared to functional genes. Thus if all of the *GCKR* genes in Laurasiatheria are pseudogenes, then the intact *GCKR* sequences from dog, panda, horse and pig should have accumulated more mutations than those of other mammals that have functional *GCKR* genes, such as rodents and humans [11], [12], [19]–[23]. To test this possibility we first constructed a phylogenetic tree (Figure 3) of the mammalian GCKR protein sequences from species that have full-length sequences, rooted with the sequence from the Tasmanian devil, a marsupial outgroup for placental mammals [47]. As shown in Figure 3, the dog, panda, horse and pig *GCKR* sequences may actually show less protein sequence evolution than other mammals, especially rodents, which is certainly not a pattern consistent with accelerated evolution that would be expected of pseudogenes. Similar conclusions are drawn from phylogenies generated using more distantly related outgroups, including fish, amphibians and reptiles, where the dog and elephant GCKR proteins sequences do not accumulate greater numbers of amino acid substitutions compared to human, mouse or Tasmanian devil (Figure 4). To confirm that there was no acceleration in the rate of amino acid substitution in the GCKR sequences of species within Lauasiatheria compared to other placental mammals we conducted relative rate tests [42]. As shown in Table 4, when the dog, panda, horse, and pig sequences were tested against the human sequence, no significant difference in the number of amino acid substitutions was seen on either lineage. When the mouse sequence was used, a significant difference in the number of substitutions was seen, but it was the mouse sequence, not the potential pseudogene sequences, which had accumulated the greater number of amino acid substitutions (Table 4). If the rat sequence was used instead of the mouse, similar results were observed (results not shown). These results indicate that the intact *GCKR* coding sequence from species within Laurasiatheria are evolving as slow, or slower, than those of other mammals thus it is extremely unlikely that they are non-functional.

**Table 4:** Relative rate tests.

Species A	Human				Mouse			
Species B	Dog	Panda	Horse	Pig	Dog	Panda	Horse	Pig
Differences in Sequence A^1^	22	23	21	18	48	52	47	44
Differences in Sequence B^1^	15	12	19	25	10	11	15	21
Chi square^2^	1.32	3.46	0.1	1.14	24.9	26.68	16.52	8.14
P	0.25	0.06	0.75	0.29	<0.01	<0.01	<0.01	<0.01

1 – Number of unique amino acid substitutions on the lineages to species A and B when the Tasmanian devil sequence was used as the outgroup.

2 – Chi square value for the expectation that an equal number of substitutions occurred on each lineage.

## Discussion

In humans, GCK has essential functions in both the liver and pancreatic beta cells, and defects at either site contribute to diabetes [3], [49]. Knockout mice have been used to define the tissue-specific roles of *GCK* [50]. Homozygous pancreatic beta cell-specific deletion of the *GCK* leads to death due to severe diabetes, while homozygous mice survive but are moderately hyperglycemia [50]. Liver-specific deletion of *GCK* leads to hyperglycemia due to defects in glucose metabolism and impacts on insulin secretion in response to glucose [50]. In the liver, GCK acts as a gatekeeper for glucose utilization, as phosphorylation of glucose by GCK drives the storage of glucose as glycogen [49]. Despite the importance of GCK function in the liver of humans, a number of vertebrate species have been reported to be deficient in GCK activity, such as cat, bat, ruminants, and birds [27], [28]. Deficiency of hepatic GCK activity is unlikely to be caused by mutations that inactivate the entire *GCK* gene as insulin secretion is not lost in these species and they do not exhibit the symptoms of diabetes that are seen when GCK is depleted from pancreatic islets of mice [50]. Liver and beta-cell expression of *GCK* is driven by two different promoters [3], [6], thus, mutations potentially could specifically inactivate the liver-specific isoform of GCK if they occur in the liver-specific first exon or prevent function of the liver-specific promoter. Indeed, this mechanism may explain GCK deficiency in bats, as the liver-specific 1^st^ exon of the *GCK* gene in bats appears to be deleted [32]. All other mammals examined, though, possess a liver-specific 1^st^ exon [32] and the sequences of this exon and liver-specific promoter do not display characteristics of being non-functional. GCKR regulates the function of GCK in the liver [15]–[17], and knockout of the mouse *GCKR* gene results in deficiency of liver GCK protein and activity [24], [25], while overexpression of GCKR in hepatocytes leads to increase in both GCK protein and enzymatic activity levels [26]. These results indicate that in liver, GCK requires GCKR for stability, and the absence of GCKR leads to GCK degradation. *GCKR* does not appear to be essential for GCK function in pancreatic beta-cells, as *GCKR* knockout mice did not show impaired insulin secretion [24], [25] and the mRNA expression ratio of *GCK*:*GCKR* is much higher in human pancreatic islets when compared with human liver [17]. As GCKR is the most important post-transcriptional regulator of GCK levels in the liver [11], [12], and vertebrate species that are deficient in hepatic GCK appear to possess a functional *GCK* gene, we raised the idea that a lack of GCKR impacts liver-specific GCK levels and activity. To test this hypothesis, we have identified and characterized *GCKR* genes from the genomes of diverse vertebrate species. We found that species that lack GCK activity had deleted or mutated *GCKR* genes, while species that have reported liver GCK activity have intact *GCKR* genes. Our results are in accord with results seen in *GCKR* knockout mice [24], [25] and with over-expression of *GCKR* [26], but further study is still needed to understand the mechanisms by which GCKR specifically regulates GCK protein levels in liver cells.

Loss of GCK activity from the liver of humans contributes to mild diabetes [4]; however, many species with limited hepatic GCK activity do not have diabetes [27]–[31]. Changes in the requirement for hepatic GCK activity may be associated with changes in diet. Ruminant animals, such as cow and sheep, acquire most of their energy from volatile fatty acids generated by fermentation in their foregut; thus, limited amounts of glucose are acquired from their diets [51]. Glucose that is needed by other tissues is produced in the liver, and its production is regulated by insulin and glucagon [51]. Therefore, ruminants do not need GCK to remove excess glucose from the circulation. Cats, like other carnivores, consume a diet containing large amounts of protein and relatively little carbohydrate [52]. As with the ruminants, glucose in carnivores is generated in the liver by a regulated process from other food sources; thus GCK may not be needed to remove excess glucose. Birds, species that have low hepatic GCK activity levels, have the highest blood glucose levels among vertebrates. With an average of 15.6 mM, or two fold higher than mammals [53], the relatively high glucose levels in birds suggests that the loss of GCKR has prevented hepatic GCK from efficiently removing excess blood glucose and may have driven additional unknown changes in glucose metabolism.

Here we have shown that the loss of hepatic GCK activity in many species is likely due to mutations in the *GCKR* gene, rather than mutations in *GCK*. Mutation of the liver-specific *GCKR* gene may have allowed these species to specifically lose GCK activity from the liver without affecting GCK activity in other glucose-sensing tissues such as pancreatic beta-cells [2], [3]. How can hepatic tissue function with the loss of GCK activity? Glucose is essential as an energy molecule by many cells in the body such as the neurons [1]; however, import of glucose is not essential for liver cells. GCK phosphorylates glucose as it enters hepatic cells, but this function is only essential if blood glucose levels vary due to diet. If glucose is not directly obtained from the diet, but rather synthesized in the liver, then blood glucose levels should be regulated by the production, rather than uptake, of glucose. As diets change, the requirements for hepatic GCK activity change, and this appears to have been achieved by the loss of *GCKR* gene function by multiple mechanisms on multiple lineages within vertebrates.

## Supporting Information

Figure S1 **Alignment of GCKR protein sequences.** Full-length and near full-length GCKR protein sequence predicted from *GCKR* genes listed in Table 1 aligned with ClustalW [40].
(DOCX)

Table S1 Genomic locations of non-mammalian *GCK* genes.
(DOC)
