# Supplementary material for: Evolution of Hepatic Glucose Metabolism: Liver-Specific Glucokinase Deficiency Explained by Parallel Loss of the Gene for Glucokinase Regulatory Protein (GCKR)
Source: PLoS One. 2013 Apr 1;8(4):e60896. doi: 10.1371/journal.pone.0060896 (PMC3613411; doi:10.1371/journal.pone.0060896)
Supplement: Figure S1 — Alignment of GCKR protein sequences. Full-length and near full-length GCKR protein sequence predicted from GCKR genes listed in Table 1 aligned with ClustalW [40]. (DOCX) [file pone.0060896.s001.docx]

Human MPGTKRFQHVIETPEPGKWELSGYEAAVPITEKSNPLTQDLDKADAENIVRLLGQCDAEI 60

Chimpanzee MPGTKRFQHVIETPEPGKWELSGYEAAVPITEKSNPLTQDLDKADAENIVRLLGQCDAEI 60

Gorilla MPGTKRLQHVIETPEPGKWELSGYEAAVPITEKSNPLTQDLDKADAENIVRLLGQCDAEI 60

Orangutan MPGTKRFQHVIETPEPGKWELSGYEAAVPITEKSNPLTQELDKADAENIVRLLGQCDAEI 60

Gibbon MPGTKRFQHVIETPEPGKWELSGYEAAVPITEKSNPLTQDLDKADAENIVRLLGQCDAEI 60

Macaque MPGTKRFQHVIETPEPGKWELSGYEAAVPITEKSNPLTQDLDKADAEEIVRLLGQCDAEI 60

Baboon MPGTKRFQHVIETPEPGKWELSGYEAAVPITEKSNPLTQDLDKADAEEIVRLLGQCDAEI 60

Mouse_lemur MPATKRFQHVIETPEPGKWELSGYEAAVPITEKSNPLTQDLDKADAEKIVQLLGQCDAEI 60

Mouse MPSTKRYQHVIETPEPGEWELSGYEAAVPITEKSNPLTRNLDKADAEKIVQLLGQCDAEI 60

Rat MPGTKRYQHVIETPEPGEWELSGYEAAVPITEKSNPLTRNLDKADAEKIVKLLGQCDAEI 60

Chinese_hamster MPGTKRYQHVIETPEPGEWELSGYEAAVPITEKSNPLTQNLDKADAEKIVQLLGQCDAEI 60

Kangaroo_rat MPGTKRXXXXXXXXXXX-XXXSGYEAAVPITEKSNPLTRDLDKADAKKIVELLGQCDAEI 59

Squirrel MPGTKRFQHVIETPEPGEWELSGYEAAVPITEKSNPLTRDLDKADVEKIVRLLGQCDAEI 60

Guinea_pig MPGTKRFQHVIETPEPGEWELSGYEAAVPITEKSNPLTRDLDKANAEKIVELLGQCDAEI 60

Rabbit MPGTKRFQHVIETPEPGEWELSGYEAAVPITEKSNPLTRDLDKADAERIVQLLGQCDAEI 60

Pig MPGTKRFQHVIETPEPGKWELSGYEAALPITEKSNPLTQDLDKADAKEIVRLLGQCDAEI 60

Horse MPGTKRFQHVIETPEPGKWELSGYEAALPITEKSNPLTRDLDKADAKQIVQLLGQCDAEI 60

Dog MPSTKRFQHVIETPEPGKWELSGYEAALPITEKSNPLTQDLDKADAEQIVRLLGQCDAEI 60

Panda MPGTKRFQHVIETPEPGKWELSGYEAALPITEKSNPLTQDLDKADAEQIVRLLGQCDAEI 60

Elephant MPGTKGFQHVIKTPEPGKWELSGYEAALPITEKSNPLTQDLDKADAQKIVQLLGQCDAEI 60

Tasmanian_devil MLGTKRFQHVIETPEPGKWELSGYEASLPITEKSNPLTQDLDKANAEQIVQLLGQCDAEI 60

Painted_Turtle MRGTKKYRHVIETPDSGKWELAGYEATLPISEKSNPITRELDKADPVQIVQLLKECDAEI 60

Xenopus_laevis MRGTRKYQHVIETPDPGKWELAGYEESLPISEKSNPMTRELDKADPSQLVQLLRDCDAEI 60

Medaka -----PATREHPVPTP-AWNKSNYEPSLPVSEKSNPFTRDIDRASPSGIVGMLEACDAQM 54

Stickleback -----------------------YEPSLPVSEKSNPLTRDMDRASAKCIVRMLQACDAQM 37

Fugu -WRGSTDSCVMKGPDR-TWESPDYEPSLPVSEKSNPLTRDIDQASAIGIVRMLQACDGQM 58

Spotted_gar MLGSRKHNHIIETPDTGKWQLAGYEASLPLTEKSNPITKEIDKADPLQIVHLLKLCDAEI 60

Lamprey -------------------EMAALEDLVPHTERANPRTHGLDTASPSQLVQLLQECDLEI 41

* :* :*::** *: :* *. :* :* ** ::

Human FQEEG--QALSTYQRLYSESILTTMVQVAGKVQEVLKEPDGGLVVLSGGGTSGRMAFLMS 118

Chimpanzee FQEEG--QALPTYQRLYSESILTTMVQVAGKVQEVLKEPDGGLVVLSGGGTSGRMAFLMS 118

Gorilla FQEEG--QALPTYQRLYSESILTTMVQVAGKVQEVLKEPDGGLVVLSGGGTSGRMAFLMS 118

Orangutan FQEEG--QALPTYQRLYSESILTTMVQVAGKVQEVLKEPDGGLVVLSGGGTSGRMAFLMS 118

Gibbon FQEEG--QALPTYQRLYSESILTTMVQVAGKVQEVLKEPDGGLVVLSGGGTSGRMAFLMS 118

Macaque FQEEG--QALPTYQRLYSESILTTMAQVAGKVQEVLKEPDGGLVVLSGGGTSGRMAFLMS 118

Baboon FQEEG--QALPTYQRLYSESILTTMVQVAGKVQEVLKEPDGGLVVLSGGGTSGRMAFLMS 118

Mouse_lemur FQEEG--QGMPTYQRLYSESILTTMVQVAGKVQEVLKEPDGGLVVLSGGGTSGRMAFLMS 118

Mouse FQEEG--QIMPTYQRLYSESVLTTMLQVAGKVQEVLKEPDGGLVVLSGGGTSGRMAFLMS 118

Rat FQEEG--QIVPTYQRLYSESVLTTMLQVAGKVQEVLKEPDGGLVVLSGGGTSGRMAFLMS 118

Chinese_hamster FQEEG--QIMPTYQRLYSESVLTTMLQVAGKVQEVLKEPDGGLVVLSGGGTSGRMAFLMA 118

Kangaroo_rat FQEEG--QAIPAYQRLYSESVLTTMMQVAGKVQEVLKEPDGGLVVLSGGGTSGRMAFLMS 117

Squirrel FQEEG--QAMPTYQRLYSESVLTTMVQVAEKVQDVLKEPEEALVVLSGGGTSGRMAFLMS 118

Guinea_pig FQEEG--QAMPAYQRLYSESVLTTMVQVAEKVQEILKEPDGGLVVLSGGGTSGRMAFLMS 118

Rabbit FQEEG--QVVPTYQRLYSESVLTTMAQVAGKVQEVLKEPDGGLVVLSGGGTSGRMAFLMS 118

Pig FQEEG--QVMPTYQRLYSESTLTTMVQVAGKVQEVLKEPEGGLVVLSGGGTSGRMAFLMS 118

Horse FQEEG--QAMPTYQRLYSDSVLTTMVQVAGKIQEVLKDPEGGLVVLSGGGTSGRMAFLMS 118

Dog FQEEG--QIMPTYQRLYSESVLTTMVQVAGKVQEVLKEPEGGLVVLSGGGTSGRMAFLMS 118

Panda FQEEG--QVMPTYQRLYSESVLTTMVQVAGKVQEVLKEPEGGLVVLSGGGSSGRMAFLMS 118

Elephant FQEEG--QTMPTYQRLYSESILTTMVQVGGKVQEVLKDPEGGLVVLSGGGTSGRMAFLMS 118

Tasmanian_devil FQEEG--QIMPAYQRLYSESILTTMVHVAGKVHEVLKEPEGGLVVLSGGGTSGRMAFLMS 118

Painted_Turtle FQEED--EALLNYTRLYSESVLKTMIDIINKVQEVLKDPDNTLIVLSGGGTSGRLAFLIA 118

Xenopus_laevis FQEED--ENLIHYHRLYSESVLKTMGDVAKRVQEVLKNPDDSLVVLSGCGTSGRLALLLA 118

Medaka FRQD----RGTNYQRLFDEQVVTTLTEVAKRVELILKDPQDSLVVLSGCGTSGRLAFFMA 110

Stickleback FQDG----TGTTYQRLLSEPVLETLTEVAKKVEHILKHPRDSLIVLSGCGTSGRLAFLMA 93

Fugu FEED----TAVKYQRLLSKQVVQTLVEVAKRVELILKDPQDSLVVLSGCGTSGRIAFLVA 114

Spotted_gar FQEEG--ETVINYQRLYSESVLQTLIDVAKKMEDILKDPEHSLVVLSGCGTSGRLAYLLV 118

Lamprey FQRDDKATAAEEHMTLFGESTIRLMTDVAAQAKIILENPENNAIVLSGCGTSGRLAFLVA 101

*. : * .. : : .: : . :*:.* :**** *:***:* ::

Human --VSFNQLMKGLGQKPLYTYLIAGGDRSVVASREGTEDSALHGIEELKKVAAGKKRVIVI 176

Chimpanzee --VSFNQLMKGLGQKPLYTYLIAGGDRSVVASREGTEDSALHGIEELKKVAAGKKRVIVI 176

Gorilla --VSFNQLMKGLGQKPLYTYLIAGGDRSVVASREGTEDSALHGIEELKKVAAGKKRVIVI 176

Orangutan --VSFNQLMKGLGQKPLYTYLIAGGDRSVVASREGTEDSALHGIEELKKVAAGKKRVIVI 176

Gibbon --VSFNQLMKGLGQKPLYTYLIAGGDRSVVASREGTEDSALHGIEELKKVASGKKRVIVI 176

Macaque --VSFNQLMKGLGQKSLYTYLIAGGDRSVVASREGTEDSALHGIEELKKVAAGKKRVIVI 176

Baboon --VSFNQLMKGLGQKSLYTYLIAGGDRSVVASREGTEDSALHGIEELKKVAAGKKRVIVI 176

Mouse_lemur --VSFNQLMKGLRQKPLYTYLIAGGDRSLVASREGTEDSAMHGIEELKKVAAGKKKVIVI 176

Mouse --VSFNQLMKGLGQKPLYTYLIAGGDRSVVASRERTEDSALHGIEELKKVAAGKKRVVVI 176

Rat --VSFNQLMKGLGQKPLYTYLIAGGDRSVVASREQTEDSALHGIEELKKVAAGKKRVVVI 176

Chinese_hamster --VSFNQLMKGLGQKPLYTYLIAGGDRSVVASREQTEDSALHGVEELKKVAAGKKRVIVI 176

Kangaroo_rat --VSFNQLMKGLGQKPLYTYLIAGGDRSVVASRERTEDSALHGIEELKKVAAGKKKVIVI 175

Squirrel --VSFNQLMKGLGQKPLYTYLIAGGDRSVVASRERTEDSALHGIEELKKVTAGKKRVIVI 176

Guinea_pig --VSFNQLMKGLGQKPLYTYLIAGGDRSVVASRERTEDSALHGIEELKKVAAGRRRVIVI 176

Rabbit --VSFNQLMRGLGQKPLYTYLIAGGDRSVVASREGTEDSALHGIEELKKVAAGKKRVIVI 176

Pig --VSFNQLMRGLGQKPLYTYLIAGGDRSVVASREGTEDSALHGIEELKKVAAGKKRVIVI 176

Horse --VSFNQLMKGLGQKPLYTYLIAGGDRSVVASREGTEDSALHGIEELKKVAAGKKRVIVI 176

Dog --VSFNQLMKGLGQKPLYTYLIAGGDRSVVASREGTEDSALHGIEELKKVAAGKKRVIVI 176

Panda --VSFNQLMKGLGQKPLYTYIIAGGDRSVMASREGTEDSALHGIEELKKVAAGKKRVIVI 176

Elephant --VSFNQLMKGLGQKPLYTYLIAGGDRSVVASREGTEDSALHGIEELKKVAAGKKRVIVI 176

Tasmanian_devil --VSFNQLMKGLGQKPLYTYLIAGGDRSVVASREGTEDSALHGIEELKKVTAGKKRVIVI 176

Painted_Turtle --VSFNKLLKGLGQLPRYTYIIAGGDXXXXXXX--------------------------- 149

Xenopus_laevis --NSFNGLLKGLHKTPCYCYIMSGGDRSIVTSQESSEDNPQLGAQELEKVCEGKKNVLFI 176

Medaka VKSRFNEALKQLNRTLVYSYIIAGGDSALLSSQEAPEDDPELGMLSLQKVCEAKKKVLFI 170

Stickleback --SAFNRALRELDHGLVYSYIIAGGDRALLSSQEAPEDDAELGVLSLQRACEGQKSVLFI 151

Fugu --SRFSRRLKDVNQSSVYSYIIAGGDRALLSSQEAPEDDAKLGRLMLKEACEGKKRVVFI 172

Spotted_gar --TSFNRLLKGLQKKHNYDYIIAGGDKALLTSQEAPEDNPYLGAEELEKVCAGKKQVLFI 176

Lamprey --KSFNKLLQDCGRSPCYTYVIAGGDKALITSQEAQEDQPELGSQQLREVTMDKKMILFI 159

*. :: : * *:::***

Human GISVGLSAPFVAGQMDCCMNNTAVFLPVLVGFNPVSMARNDPIEDWSSTFRQVAERMQKM 236

Chimpanzee GISVGLSAPFVAGQMDYCMNNTAVFLPVLVGFNPVSMARNDPIEDWSSTFRQVAERMQKM 236

Gorilla GISVGLSAPFVAGQMDYCMNNTAVFLPVLVGFNPVSMARNDPIEDWSSTFRQVAERMQKM 236

Orangutan GISVGLSAPFVAGQMDYCMNNTAVFLPVLVGFNPVSMARNDPIEDWSSTFRQVAERMQKI 236

Gibbon GISVGLSAPFVAGQMDYCMNNTAVFLPVLVGFNPVSMARNDPIEDWSSTFRQVAERMQKM 236

Macaque GISVGLSAPFVAGQMDYCMNNTAVFLPVLVGFNPVSMARNDPIEDWSSTFRQVAERMQKM 236

Baboon GISVGLSAPFVAGQMDYCMNNTAVFLPVLVGFNPVSMARNDPIEDWSSTFRQVAERMQKM 236

Mouse_lemur GISVGLSAPFVAGQMDYCMDNTAVFLPVLVGFNPVSMARNDPIEDWSSTFRQVAERMQKM 236

Mouse GISVGLSAPFVAGQMDYCMDNTAVFLPVLVGFNPVSMARNDPIEDWRSTFRQVAERMQKM 236

Rat GISVGLSAPFVAGQMDYCMDNTAVFLPVLVGFNPVSMARNDPIEDWRSTFRQVAERMQKM 236

Chinese_hamster GISVGLSAPFVAGQMDYCMDNTAVFLPVLVGFNPVSMARNDPIEDWRSTFRQVAERMQKM 236

Kangaroo_rat GISAGLSAPFVAGQMDYCMDNTAVFLPVLVGFNPVSMARNDPIEDWNSTFRQVAERMKKM 235

Squirrel GISVGLSAPFVAGQMDYCMDNTDVFLPVLVGFNPVSMARNDPIEDWSSTFRQVAERMQKM 236

Guinea_pig GISVGLSAPFVAGQMDYCMDNTAVFLPVLVGFNPVNMARNDPIEDWRSTFRQVAERMQKL 236

Rabbit GISVGLSAPFVAGQMDYCMDNTAVFLPVLVGFNPVSMARNDPIEDWSSTFRQIAERMQKM 236

Pig GISVGLSAPFVAGQMDYCMDNPAVFLPVLVGFNPVSMARNDPIEDWSSTFRQIAERMQKL 236

Horse GISVGLSAPFVAGQMDYCMDNPAVFLPVLVGFNPVSMARNDPIEDWSSTFRQIAERMQNL 236

Dog GISVGLSAPFVAGQMDYCMDNPAIFLPVLVGFNPVNMARNDPIEDWSSTFRQIAERMQKL 236

Panda GISVGLSAPFVAGQMDYCMDNPAIFLPVLVGFNPVNMARNDPIEDWSSTFRQIAERMQKL 236

Elephant GISVGLSAPFVAGQMDYCMDNPAVFLPVLVGFNPVSMARDDPIEDWSSTFRQVAERMKKL 236

Tasmanian_devil GISVGLSAPFVAGQMDFCMNNSDIFLPVLVGFNPVNMARNDPIEDWNSTFREIAQRMQKL 236

Painted_Turtle -------APFVAGQLDFCMNNLDIFLPVLVGFNPVSMARNDKIEGWHSTFRQVAERMQKL 202

Xenopus_laevis GISCGLSAPFIAGQLDFCMRHLDVYLPVLVGFNPVSMARNERIEGWHSSFRQVAERLQTL 236

Medaka GVSCGLSAPFVAGQLDFCLQHPEVYTPVLLGFNPTHQARDEPIPGCIFTFHSVAQRLQEL 230

Stickleback GISCGLSAPFVAGQLDFCLQHPEVYTPVLVGFNPTHQARDERIPGCSFTFRSVVQRMQEL 211

Fugu GVSCGLSAPFVAGQLDFCLRHPEVFTPVLVGFNPAHQARDEPIEGCTFTFRSVVQRMLEL 232

Spotted_gar GISCGLSAPFIAGQLDFCMNNLNVFTPVLVGFNPTNMARNEIIEGWHLTFYQVAERMQEL 236

Lamprey GITCGLSAPFVAGQLDMCLADPGRFTAVLLGFNPLSLARTTKVEGWHSSFAQVAERIHSR 219

***:***:* *: . : .**:**** ** : . :* .:.:*:

Human QE-KQKAFVLNPAIGPEGLSGSSRMKGGSATKILLETLLLAAHKTVDQGIAASQRCLLEI 295

Chimpanzee QE-KQKAFVLNPAIGPEGLSGSSRMKGGSATKILLETLLLAAHKTVDQGIAASQRCLLEI 295

Gorilla QE-KQKAFVLNPAIGPEGLSGSSRMKGGSATKILLETLLLAAHKTVDQGIAASQRCLLEI 295

Orangutan QE-KQKAFVLNPAIGPEGLSGSSRMKGGSATKILLETLLLAAHKTVDQGIAASQRCLLEI 295

Gibbon QE-KQKAFVLNPAIGPEGLSGSSRMKGGSATKILLETLLLAAHKTVDQGIAASQRCLLEI 295

Macaque QE-KQTAFVLNPAIGPEGLSGSSRMKGGSATKILLETLLLAAHKTVDQGIAASQRCLLEI 295

Baboon QE-KQKAFVLNPAIGPEGLSGSSRMKGGSATKILLETLLLAAHKTVDQGIAASQRCLLEI 295

Mouse_lemur QE-KQKAFVLNPAVGPEGLSGSSRMKGGSATKILLETLLLAAHKTVDRGIEASQRCLLEI 295

Mouse QE-KQEAFVLNPAIGPEGLSGSSRMKGGSATKILLETLLLAAHKTVDQGVVSSQRCLLEI 295

Rat QE-KQEAFVLNPAIGPEGLSGSSRMKGGGATKILLETLLLAAHKTVDQGVVSSQRCLLEI 295

Chinese_hamster QE-RQEAFVLNPAVGPEGLSGSSRMKGGSATKILLETLLLVAHKTVDQGVVASQRCLLEI 295

Kangaroo_rat QE-KQEAFVLNPAVGPEGLSGSSRMKGGSATKILLETLLLAAHKTVDQGIATSQRYLLEI 294

Squirrel QE-KQEAFVLNPAVGPEGLSGSSRMKGGSATKILLETLLLAAHETVDCGIAASQRCLLEI 295

Guinea_pig QE-KQEAFVLNPAVGPEGLSGCSRMKGGSATKILLETLLLAAHKTVDRGIAASQRCLLEI 295

Rabbit QE-RQEAFVLNPAVGPEGLSGSSRMKGGSATKVLLETLLLAAHKTVDRGIAASPKCLLEI 295

Pig QE-KQEAFVLNPAIGPEGLSGCSRLKGGSATKVLLETLLLAAHKTVDRGIEASPRCLLEI 295

Horse QE-KQEGFVLNPAIGPEGLSGSSRMKGGSATKILLETLLLAAHKTVDRGIATSQRCLLEI 295

Dog QE-KQEAFVLNPAIGPEGLSGSSRMKGGSATKILLETLLLAAHKTVDRGIAASQRCLLEI 295

Panda QE-KQEAFVLNPAIGPEGLSGSSRMKGGSATKILLETLLLAAHKTVDRGIAASQRCLLEI 295

Elephant QE-KQEAFVLNPAIGPEGLSGSSRMKGGSATKILLETLLLAAHKTVNRGIAASQRCLLEI 295

Tasmanian_devil QE-KQKAFVLNPAIGPEGLSGSSRMKGGSATKILLETLLLAAHHTVERDMGARRSCLLEI 295

Painted_Turtle QE-SHKAFILNPAVGPEGISGSSRMKGGSATKILLETLLLAAHKTVSKDTDISEKCLLEI 261

Xenopus_laevis HD-SQKGFILNPAVGPEGVSGSSRMKGGSATKILLETLLLVAHKAESN-VPVTEKCLLEI 294

Medaka SQ-RRKAFLINPAVGPEAISGSSRMKGGSATKIVLEVVLSAAHAAAFSHKSVASECILQH 289

Stickleback AK-RGGAFVVNPAVGPEAISGSSRMKGGSATKILLEAVLSAAHAAAFADTPITPMGIRED 270

Fugu AQ-SQRAFIISPAVGPEAISGSSRMKGGSATKILLEVILSTAQAAAFSRAPLTETVTLQY 291

Spotted_gar QK-NYRAFILNPAVGPEAITGSSRMKAGSATKIMLEILFLAAHRAAFSNKEITANGILDC 295

Lamprey SISTATSFLLNPLVGAEALTGSTRMKGGSATKVVLETVFLAAHTALNAGVAVSPSCVREC 279

.*::.* :*.*.::*.:*:*.*.***::** :: .*: : :

Human LRTFERAHQVTYSQSPKIATLMKSVSTSLEKKGHVYLVGWQTLGIIAIMDGVECIHTFG- 354

Chimpanzee LRTFERAHQVTYSQSPKIAALMKSVSTSLEKKGHVYLVGWQTLGIIAIMDGVECIHTFG- 354

Gorilla LRTFERAHQVTYSQSPKIATLMKSVSTSLEKKGHVYLVGWQTLGIIAIMDGVECIHTFG- 354

Orangutan LRTFERAHQVTYSQSPKIATLMKSVSTSLEKKGHVYLVGWQTLGIIAIMDGVECIHTFG- 354

Gibbon LRTFERAHQVTYSQSPKIATLMKSISTSLEKKGHVYLVGWQTLGIIAIMDGVECIHTFG- 354

Macaque LRTFERAHQVTYSQSPKIATLMKSVSTSLEKKGHVYLVGWQTLGIIAIMDGVECIHTFG- 354

Baboon LRTFERAHQVTYSQSPKIATLMKSVSTSLEKKGHVYLVGWQTLGIIAIMDGVECIHTFG- 354

Mouse_lemur LWTFERAHQVTYSQSARIATLMKQVSTSLERKGRTYLVGWQTLGIIAIMDGVECIHSFG- 354

Mouse LRTFERAHQVTYSQSSKIATLTKQVGISLEKKGHVHLVGWQTLGIIAIMDGVECIHTFG- 354

Rat LRTFERAHQVTYSQSSKIATLMKQVGISLEKKGRVHLVGWQTLGIIAIMDGVECIHTFG- 354

Chinese_hamster LRTFERAHQVTYSQSSKIATLMKQVSTSLEKKGRVYLVGWQTLGIIAIMDGVECIHTFG- 354

Kangaroo_rat LRTFERAHQVTYSQSSKIATLMKQVSTSLEKKGRVHLVGWQTLGIIAIMDGVECIHTFG- 353

Squirrel LRTFERAHQVTYSQNSKIATLMKQVSTSLEKKGRVHLVGWQTLGIIAIMDGVECIHTFG- 354

Guinea_pig LRTFERAHKVTYSQSSKIATLMKQVSTSLEKKGRVHLLGWQTLGIIAIMDGVECIHNFG- 354

Rabbit LRTFERAHQVTYSQSPKIAALMKQVSSSLKKKGRVHLVGWQTLGIIAIMDGVECIHTFG- 354

Pig LRTFERAHQVTYSQSAKIAALMKQASTSLEKKGRVHLVGWQTLGIIAIMDGVECIHSFG- 354

Horse LRTFERAHQVTYSQSPKIAALVKQASTSLEKKGQVYLVGWQTLGIIAIMDGVECIHTFG- 354

Dog LRTFERAHQVTYSQSTKIAALMKQASSSLEKKGRVYLVGWQTLGIIAIMDGVECIHTFG- 354

Panda LRTFERAHQVTYSQSPKIAALMKQASTSLEKKGRVYLVGWQTLGIIAIMDGVECIHTFG- 354

Elephant LRTFERAHQVTYSQSPKIAALMKQASTSLEKKGRVCLVGWQTLGIIAIMDGVECIHTFG- 354

Tasmanian_devil LRTFERAHKVTYSQGPKIASLMKQASTSLQKKGRVYLVGWQTLGIIAIMDGVECIHTFG- 354

Painted_Turtle LRTYERAHKVTYAQSKKIAALVKQAGTSLQKKACVYMVGWHTLGIIAIMDGAECIPTFG- 320

Xenopus_laevis LRTYERAHKVTYSQSKKIAALMKQTATSLQKKGHLYILGWGTLGLVGIMDAVECVPTYQ- 353

Medaka MREYERAVEVTYQQKEGIAAVVEAAGRSLLNEGSVCYLGWGPMAILGLIDASECNPTFG- 348

Stickleback MSVHEKTLEITYAQAKGIGALLEAAGQSLRRGGRLCYLGWGSLGTLGLVDASECVPTFG- 329

Fugu LLAYKNAVDVTYSRTEQISTLVEAAGKSLRCGGRVCYLGWGTLAVLGLIDASECNPTFG- 350

Spotted_gar LQAYERVHKVTYSQSKKISVLVNQVGESLQKGGHVYYIGWQTLGVMGIIDASECPPTFG- 354

Lamprey LLSYEAVHKTTYACSPSIAELVHRCGTSLQQAGHLYYLGWGTLGIVAMIDASECPPTFGA 339

: .: . . ** *. : . . ** . :** .:. :.::*. ** .:

Human ADFRDVRGFLIGDHSDMFNQKAELTNQGPQFTFSQEDFLTSILPSLTEIDTVVFIFTLDD 414

Chimpanzee ADFRDVRGFLIGDHSDMFNQKAELTNQGPQFTFSQEDFLTSILPSLMEIDTVVFIFTLDD 414

Gorilla ADFRDVRGFLIGDHSDMFNQKAELTNQGPQFTFSQEDFLTSILPSLTEIDTVVFIFTLDD 414

Orangutan ADFRDVRGFLIGDHSDMFNQKAELTNQGPQFTFSQEDLLTSILPSLTEIDTVVFIFTLDD 414

Gibbon ADFQDVRGFLIGDHSDMFNQKAELTNQGPQFTFSQEDFLTSILPSLTEIDTVVFIFTLDD 414

Macaque ADFRDVRGFLIGDHSDMFNQKAELTNQGPQFTFSQEDFLTSILPSLTEIDTVVFIFTLDD 414

Baboon ADFRDVRGFLIGDHSDMFNQKAELTNQGPQFTFSQEDFLTSILPSLTEIDTVVFIFTLDD 414

Mouse_lemur ADFRDIRGFLIGDHSDMFNQKAELTNQGPQFSFSQEDFLTSILPTLTDIDTVVFIFTLDD 414

Mouse ADFRDIRGFLIGDHNDMFNQKDELSNQGPQFTFSQDDFLTSVLPSLTEIDTVVFIFTLDD 414

Rat ADFQDIRGFLIGDHSDMFNQKDELTNQGPQFTFSQDDFLTSILPSLTETDTVVFIFTLDD 414

Chinese_hamster ADSQDVRGFLIGDHNDMFNQKAELTNQGPQFTFSQDDFLTSILPSLVEIDTVVFVFTLDD 414

Kangaroo_rat ADFRDIRGFLIGDHSDMFNQKAELTNQGPQFSFSQEDFLTSILPSLKEIDTVIFIFTLDD 413

Squirrel ADFRDVRGFLMGDHSDMFNQKAELTNQGPQFSFSQEDFLTSILPSITEIDTVLFIFTLDD 414

Guinea_pig ADFRDIRGFLIGDHSDMFNQKAELINQGPQFTFSQEDFLTSVLPSLTETDTVIFIFTLDD 414

Rabbit ADFRDIRGFLMGDHSDMFNQKAELTNQGPQFTFSQEDFQTSILPSLTEIDTVVFIFTLDD 414

Pig ADFRDIRGFLIGDHSDMFNQKAELINQGSHFTFSQEDFLTSILPALTEVDTVVFIFTLDD 414

Horse ADFQDVRGFLIGDHSDIFNQKADLINQGPQFSFSQEDFLTSILPSLKEIDTVVFIFTLDD 414

Dog ADFQDVRGFLIGDHSDMFNQKAELINQGPQFCFSQEDFLTSILPSLTEIDTVVFVFTLDD 414

Panda ADFRDVRGFLIGDHSDMFSQKAELINQGPQFSFSQEDFLTSILPSLTEIDTVVFIFTLDD 414

Elephant ADYQDVRGFLIGDHSEMFNQKAELINQGPRFSFSQEDFQTSVLPSLTENDTVIFIFTLDD 414

Tasmanian_devil ADFQDVRGFLIGDHSDMFNQKEEIVAQGPQFSFSQEDFLTSILPSLTEKDTVLFIFTLDD 414

Painted_Turtle AXXX----------------------XGPQVAFSQEDFVKMILPSLTELDTVLFLFTLDD 358

Xenopus_laevis ADWRDVRGFITGGYHSIENKEGDLSSLGPQFSISHEDFVKNVLPSVSETDTVLLIFTLDD 413

Medaka ADYTDIRGFIRGGYKDLNNNEGPLSSLGPEFCIGHEDFLQHVLPGLADKDTVLLIYSQSD 408

Stickleback AGCEDVRGFISGGYRALNNNEGPLADKGPDFSIAHQDFLHQVLPSLTDRDVVLLLYTHAD 389

Fugu ADYEDIRGFIAGGYVELDNNEGPLTSLGPDFSISHEEFFRSVLPSLTDRDTVLLLYTHSD 410

Spotted_gar ADVGDVRGFINNGYKEMDNKEGNIASLGPEFCIAHDDFVRNILPHVNDNDTVLFLFTLDD 414

Lamprey EDINDVRAFLHGGYQTLGIKEGDVSSKGQQFFLSHEDFKKNILPCLTDWDTVILIFTGDD 399

* . :.:::: :** : : *.*::::: *

Human NLTEVQTIVEQVKEKTNHIQALAHSTVGQTLLIPLKKLFPSIISIT--WPLLFFEYEGNF 472

Chimpanzee NLTEVQTIVEQVKEKTNHIQALAHSTVGQTLPIPLKKLFPSIIRIT--WPLLFFEYEGNF 472

Gorilla NLTEVQTIVEQVKEKTNHIQALAHSTVGQTLPIPLKKLFPSIISIT--WPLLFFEYEGNF 472

Orangutan NLMEVQTIVEQVKEKTSHIQALAHSTVGQTLPIRLKKLFPSIISIT--WPLLFFEYEGNF 472

Gibbon NLTEVQTLVEQVKEKTSHIQALAHSTVGQTLPIPLKRLFPSIISIT--WPLLFFEYEGNF 472

Macaque NLTEVQTIVEQVKEKTSHIQALAHSTVGQNLPIPLKKLFPSVISIT--WPLLFFEYEGNF 472

Baboon NLTEVQTIVEQVKEKTSHIQALAHSTVGQTLPIPLKKLFPSIISIT--WPLLFFEYEGNF 472

Mouse_lemur NLTEVQTLVEQVKEKTTNIQALVHSTVGQSLPTPLKKLFPSIISIT--WPLLFFEYEGNF 472

Mouse NLAEVQALAERVREKSWNIQALVHSTVGQSLPAPLKKLFPSLISIT--WPLLFFDYEGSY 472

Rat NLTEVQALAERVREKCQNIQALVHSTVGQSLPAPLKKLFPSLISIT--WPLLFFDYEGTY 472

Chinese_hamster NLTEVQALVEQVRKKTTSIQALVHSTVGQSLPAPLKRLFPSIISIT--WPLLFFEYEGSY 472

Kangaroo_rat NLPEVQTLVEQVKEKTANIQALVHSTVGQSLPLPLKKLFPSIIHIM--WPLLFFEYEGTF 471

Squirrel NLMEVQTLVEQVKEKTINIQALAHSTVGQSLPTPLKKLFPSIISIT--WPLLFFEYEGNF 472

Guinea_pig NLTQVQTLVEQVKEKTSSIQALTHSTVGQSLPAPLKKLFPSIINIM--WPLLFFEYEGNF 472

Rabbit NLAEVQTLVEQVKEKTSNIQALAHSTVGQSLPSPLKKLFPSIISIT--WPLLFFEYEGNF 472

Pig NLAEVQTLVEQVKEKTSNIQALAHGTVGQSLPTPLKKLFPSIISIT--WPLLFFEYEGNF 472

Horse NLTEVQTLVEQVKEKTSHIQALAHSTVGQSLPTTLKKLFPSIISIT--WPLLFFEYEGNF 472

Dog NLTEVQTLVEQVKEKTANIQALAHSTVGQSLPTPLKKLFPSIISIM--WPLLFFEYEGNF 472

Panda NLTEVQTLVEQVKEKTANIQALAHSTVGQTLPTPLKKLFTSIISIT--WPLLFFEYEGNF 472

Elephant NLTEVQTLAEQVKEKTTNIQALAHSTVGQSLPTLLKKLFPSIISIT--WPLLFFEYEGNF 472

Tasmanian_devil NLTEVETLVEQVKKKTPNIQALAHSTVGQYLPASLKKLFPSMISIT--WPLLFFDYAGNF 472

Painted_Turtle DLAEVEKLVVQVKEKTSNVQALSHATVGQYLPASLKKLFPSIMSIM--WPILFLEYEGNF 416

Xenopus_laevis DLNQIEKLVALVKEKTSNIQVICHATAGQYLPNSLKKTIPSIIGLT--WPILFLEYEGAF 471

Medaka DLHEVLNLGRRVREKTPNLHAVHHRTDSDSAETPQQKSLRLLAS----FFSWIVFLFPQI 464

Stickleback RVGEVAELARAAREKTSNVHAVYHRVDGDTAAAVQQVCKTKLVYINFQIFLFLVFYKKKF 449

Fugu DLGEVAALARRVRERTSSLHAIYHRGGGDNAATDRQVTVCPPACP---SVCLSVIYLPMF 467

Spotted_gar DLSEIDKLATQISANTSNLHAVAHSTAGHYVPENVKKSFASIISIT--WPIVFSEYEGCF 472

Lamprey DQLEVRCFAETIRARSPHISAIVHASSEQHLRGELESLFATIVKVT--WPTISSHKMDHY 457

:: : . : .: * . :

Human IQKFQRELSTKWVLNTVSTGAHVLLGKILQNHMLDLRISNSKLFWRALAMLQRFSGQSKA 532

Chimpanzee IQKFQRELSTKWVLNTVSTGAHVLLGKILQNHMLDLRISNSKLFWRALAMLQRFSGQSKA 532

Gorilla IQKFQRELSTKWVLNTVSTGAHVLLGKILQNHMLDLRISNSKLFWRALAMLQRFSGQSKA 532

Orangutan IQKFQRELSTKWVLNTVSTGAHVLLGKILQNHMLDLRISNSKLFWRALAMLQRFSGQSKA 532

Gibbon IQKFQRELSTKWVLNTVSTGAHVLLGKILQNHMLDLRISNSKLFWRALAMLQRFSGQSKA 532

Macaque VQKFQRELSTKWVLNTVSTGAHVLLGKILQNHMLDLRISNSKLFWRALAMLQRFSGQSKA 532

Baboon VQKFQRELSTKWVLNTVSTGAHVLLGKILQNHMLDLRISNSKLFWRALAMLQRFSGQSKA 532

Mouse_lemur IQKFQRELSTKWVLNTVSTGAHVLLGKILQNHMLDLRISNSKLFWRALTMLQRFSGQSKA 532

Mouse VQKFQRELSTKWVLNTVSTGAHVLLGKILQNHMLDLRIANSKLFWRALAMLQRFSGQSKA 532

Rat VQKFQRELSTKWVLNTVSTGAHVLLGKILQNHMLDLRIANSKLFWRALAMLQRFSGQSKA 532

Chinese_hamster VQKFQRELSTKWVLNTVSSGAYVLLGKILQNHLLDLRIANSKLFWRALAMLQRFSGQSKA 532

Kangaroo_rat VQKFQRELSTKWVLNTVSTGAHVLLGKSLHNYMLDLRIANSKLFWRALAMLQRFSGQSKA 531

Squirrel IQKFQRELSTKWILNTVSTGAHVLLGKILQNYMLDLRIGNSKLFWRALAMLQRFSGQSNA 532

Guinea_pig VQKFQRELSTKWVLNTVSTGAHVLLGKILQNHMLDLRIANSKLFWRALAMLQRFSGQSKA 532

Rabbit VQKFQRELSTKWVLNTVSTGAHVLLGKILQNHMLEIRISNSKLFWRALAMLQRFSGQSKA 532

Pig IQKFQHELSTKWVLNTVSTGAHVLLGKILQNHTLDLRIRNSKLFWRALSMLQRFSGQPKA 532

Horse IQKFQHELSTKWVLNTVSTGAHVLLGKILQNHMSDLRIRNSKLFWRALAMLQRFSGQPKA 532

Dog IQKFQHELSTKWILNTVSTGAHVLLGKILQNHMLDLRIRNSKLFWRALAMLQRFSGQPKA 532

Panda IQKFQHELSTKWVLNTVSTGAHVLLGKILQNHMLDLRIRNSKLFWRALAMLQRFSGQPKA 532

Elephant IQKFQHELSTKWVLNTVSTGAHVLLGKILQNHMLDLRIGNSKLFWRALAMLQRFSGQPKA 532

Tasmanian_devil IQKFQRELSTKWVLNTVSTGAHVLCGKILHNHMLDLRLRNSKLFWRGLAMLQRFSEQPKA 532

Painted_Turtle IQKFQRELSTKWILNTVSTGAHVLKGKILHNYMVDLRISNSKLFWRAVSILQRLTGHSQA 476

Xenopus_laevis IQKFQRELSTKWILDTVTSGAYTLRGKIFRNFMVDFKINNSKLFHRATSVLQRLTGQSQQ 531

Medaka PSFY-WELSTKLVLNAVSTGAHILKGKVYRNHMIDLQVTNSKLYRRATRLLQTLSGRAES 523

Stickleback IRFYQWELSTKLVLNAVSTGAHVLKGKIYQNYMVDVQVTNSKLYRRATRLLQKLSGCPES 509

Fugu VQEQ-WELSTKLLLNAISTGGHVLKGKVYRNHMVDVQVTNSKLYRRAARLLQKLSGHPKS 526

Spotted_gar VQKLQRELCTKWILNIISTGGHILKGKIYYNYMMDLKVSNTKLFRRALAILQRVTGQSRL 532

Lamprey LKVHKRYAIKKLIISFIFLKAITLL--IMKTVMPIARVVNDNGFTTADDVLQKFTGQPRK 515

.* ::. : . * . :: * : : . :** .: ..

Human RCIESLLRAIHFPQPLSDDIRAAPISCHVQVAHEKEQVIPIALLSLLFRCSITEAQAHLA 592

Chimpanzee RCIESLLRAIHFPQPLSDDIRAAPISCHVQVAHEKEQVIPIALLSLLFRCSITEAQAHLA 592

Gorilla RCIESLLRAIHFPQPLSDDIRAAPISCHVQVAHEKEQVIPIALLSLLFRCSITEAQAHLA 592

Orangutan RCIESLLRAIHFPQPLSDDIRAAPISCHVQVAHEKDQVIPIALLSLLFRCSITEAQAHLA 592

Gibbon RCIESLLRAIHFPQPLSDDIRAAPISCHVQVAHEKEQVIPIALLSLLFRCSITEAQAHLA 592

Macaque RCIESLLRAIHFPQPLSDDIRAAPISCHVQVAHEKEQVIPIALLSLLFRCSITEAQAHLA 592

Baboon RCIESLLRAIHFPQPLSDDIRAAPISCHVQVAHEKEQVIPIALLSLLFRCSITEAQAHLA 592

Mouse_lemur RCIDSLLQAIHFPQPLSDDIRAAPISCHVRLAHEKDQVIPVALLSLLFRCSIAEAQAHLA 592

Mouse RCIESLLQVIHFPQPLSNDVRAAPISCHVQVAHEKEKVIPTALLSLLLRCSITEAKERLA 592

Rat RCIESLLQAIHFPQPLSDDVRAAPISCHVQVAHEKEKVIPTALLSLLLRCSISEAKARLS 592

Chinese_hamster RCIESLLQAIHFPQSLSDDVRAAPISHHVQVAHEKEKVIPTALLSLLLRRPIAEAKARLA 592

Kangaroo_rat RCFESLLQAIHFPQLLSDEIRAAPISRHVHIAHEKEKVIPLALLSLLFRCSIPEALSHLA 591

Squirrel RCIESLLQAIHFPQPLSDEIRVAPISCHIQVAQEKEQVIPVALLSLLFRCSIPEAQAHLA 592

Guinea_pig RCIESLLQAIHFPQPLTDEIRAAPISTHVQAAHEKEQIIPVALLSLLFRCSIPAARAHAA 592

Rabbit RCIESLLQAIHFPQPLSDDIRAAPISRHIEVAHCKDQVIPVALLSLLFRCSIPEAQAHLA 592

Pig RCIESLLQAIHFPQPLSDDVRAAPISFHVQAADKKEQVIPIALLSLLFRCSIPEAQAHLA 592

Horse PCIESLLQVIHFPQPLSDDIRAAPISFHIQVAHEKEQVIPIALLSLLFRCSIPEAQAHLA 592

Dog RCIETLLQVIHFPQPLSDDIRASPISVHIQTAHEKEQVIPIALLSLLFRCSILEAQAHLA 592

Panda RCIETLLQVIHFPQPLSDGIRAAPISLHVQVAHEKEQVIPTALLSLLSRCSIPEAQAQLA 592

Elephant RCFESLLQVIHFPQPLSDDIRAAPISSHIQVAHEKEQVIPIALLSLLFRCSIPEAQAHLA 592

Tasmanian_devil QCIESLLKAIHFPQELSEEIREAPISRHIQVAHEKEQVIPKALLTLLFRCSIPEAEAQLA 592

Painted_Turtle RCLEGLLQTIYDPEVLSDDIRNAEQSKHIAIATEKNKVVPTALLCLLRNCSVQEAQLRLD 536

Xenopus_laevis RCTEVLLQSIYGEQTLSEQIRNTTIAGHVEAAASQDKVLPVAIVSLLRSCTIQDSRSRIN 591

Medaka ECEEALLKAIYGADKLMKDIISSDITAHTQVARNAKKVVPSALVSLLTGCALKEVESCL- 582

Stickleback RCEEALLKAVYRVDELTADITSSDIIEHAQAARSATKAVPLALVCLLSDCSLLEAGTRLE 569

Fugu RCEEALLKAIYQSEQLTSAIPLSDVRAHTAAANHRSRVVPLALILLLTNCSLKEAESRLE 586

Spotted_gar RCQEALLQAIYDTDELTDPIRTSEVTKHTQMASTKNQVVPTAVVILARNCSLTEARSRLD 592

Lamprey ECLKALLRCIYETEEISEETSNADISRHIQVIAHKEKVVPTAMVALLCDCSVTEARRLLS 575

* . **: :: : : : * : :* *:: * .:

Human AAPSVCEAVRSALAGPGQKRTADPLEILEPDVQ---- 625

Chimpanzee AAPSVCEAVRSALAGPGQKRTADPLEILEPDVQ---- 625

Gorilla AAPSVCEAVRSALAGPGQKRTADPLEILEPDVQ---- 625

Orangutan AAPSVCEAVRSALAGPGQKRTADPLEILEPDVQ---- 625

Gibbon AAPSVCEAVRSALAGPGQKRTADPLEILEPDVQ---- 625

Macaque ATPSVCEAVRSALAGPGQKRTADPLEILEPDGQ---- 625

Baboon AAPSVCEAVRSALAGPGQKRTADPLEILEPDGQ---- 625

Mouse_lemur AAPSVCEAVRSALAGPGQKRSVDSLKTLEPAVQ---- 625

Mouse AASSVCEVVRSALSGPGQKRSIQ--AFGDPVVP---- 623

Rat AASSVCEVVRSALSGPGQKRSTQ--ALEDPPACGTLN 627

Chinese_hamster AAPSVCEVVRSALSGPGQKRTTS--ETLQPGVQ---- 623

Kangaroo_rat AAPSVCEVIRSALAGPGQKRSADTLETLQPAVQ---- 624

Squirrel AALSVCEAVRSALAGPGRKRRTDSLESLQPALQ---- 625

Guinea_pig AAPSVCEAVRSALSGPGRKRSAEPREAVQPAAQ---- 625

Rabbit AASSVCEAVRSALTGPGRKRSAD---PQEPAVQ---- 622

Pig GAPSVCEAVRSALTGPGRKRNADALETLEPALP---- 625

Horse AAPSVCEAVRSALTGPGRKRSAEPLETLEPALQ---- 625

Dog AAPSVCEAVRSALTGPGRKRSTDPLEALEPALL---- 625

Panda AAPSVCEAVRSALTGPGRKRVADPLETLEPALL---- 625

Elephant TAPSVCEAVRSALTGPGRKRSADPLETLEPVLQ---- 625

Tasmanian_devil AAPSVCEAIRNALLRAGRKRHADSLDTLKSTTG---- 625

Painted_Turtle TSPSIRAAIESALNAPGRKRGADKSDSTGRRM----- 568

Xenopus_laevis SSLSIRSAIESSMNVPGRKRGAEDSESR--------- 619

Medaka -------------------------------------

Stickleback REPIVREALEACLS----------------------- 583

Fugu QQPIIREVV---------------------------- 595

Spotted_gar AHPTMREAIEACLRATGRKRTADQIAAGIHGEND--- 626

Lamprey ERRVIRDAIEHCL------------------------ 588

**Figure S1. Alignment of GCKR protein sequences.**

Full length and near full-length GCKR protein sequence predicted from *GCKR* genes listed in Table 1 were aligned with ClustalW [43].
