# Supplementary material for: Evolution of Hepatic Glucose Metabolism: Liver-Specific Glucokinase Deficiency Explained by Parallel Loss of the Gene for Glucokinase Regulatory Protein (GCKR)
Source: PLoS One. 2013 Apr 1;8(4):e60896. doi: 10.1371/journal.pone.0060896 (PMC3613411; doi:10.1371/journal.pone.0060896)
Supplement: Table S1 — Genomic locations of non-mammalian GCK genes. (DOC) [file pone.0060896.s002.doc]

**Table S1. Genomic Location of Non-Mammalian Glucokinase (GCK) Genes.**

| Species | Common name | Chromosome/Scaffold | Position | Protein ID |
| --- | --- | --- | --- | --- |
| Anolis carolinensis | Lizard | Scaffold GL343238.1 | 356,106-379,706 | ENSACAG00000011822 |
| Gallus gallus | Chicken | Scaffold AADN03010888.1 | 4796-9274 | ENSMGAT00000018556_1 |
|  |  |  |  | ENSMGAT00000020448_1 |
| Meleagris gallopavo | Turkey | GL429221.1: 1-1,152 |  | ENSMGAP00000018534 |
|  |  | GL429651.1: 1-2,330 |  | ENSMGAP00000018117 |
| Anas platyrhynchos | Duck | scaffold10397 |  | ex 8 |
|  |  | scaffold7033 |  | ex 9-10 |
| Taeniopygia guttata | ZebraFinch | Chr 22 | 129425-133088 | ENSTGUG00000003490 |
| Melopsittacus undulatus | Budgerigar | JH536464.1: 1-8,437 |  | HUMHOMP00000223366_1 |
| Xenopus tropicalis | Xenopus | Scaffold GL172666.1 | 2,537,509-2,548,106 | ENSXETG00000019003 |
| Latimeria chalumnae | Coelacanth | Scaffold JH126904.1 | 774,071-825,054 | HUMHOMG00000223366_1 |
| Gadus morhua | Cod | GeneScaffold_3225 | 103,598-107,589 | ENSGMOG00000007309 |
| Oryzias latipes | Medaka | Chr 9 | 5,058,732-5,062,498 | ENSORLG00000002010 |
| Gasterosteus aculeatus | Stickleback | groupXIII | 1,853,868-1,857,780 | ENSGACG00000004420 |
| Tetraodon nigroviridis | Tetraodon | Chr 12 | 5,886,847-5,890,080 | ENSTNIG00000015932 |
| Takifugu rubripes | Fugu | scaffold_50 | 1,273,029-1,276,196 | ENSTRUG00000016548 |
| Oreochromis niloticus | Tilapia | Scaffold GL831230.1 | 693,068-696,773 | PREONIG00000123734 |
| Danio rerio | Zebrafish | Chr 8 | 42,651,713-42,701,712 | ENSDARG00000068006 |
| Petromyzon marinus | Lamprey | Scaffold GL476526 | 302,970-319,386 | ENSPMAG00000003141 |
